# Supplementary material for: Predictors and triggers of incivility within healthcare teams: a systematic review of the literature
Source: BMJ Open. 2020 Jun 7;10(6):e035471. doi: 10.1136/bmjopen-2019-035471 (PMC7282335; doi:10.1136/bmjopen-2019-035471)
Supplement: Supplementary data [file bmjopen-2019-035471supp001.pdf]

Additional Material, Table 1: Search strategy used on Medline

**Step 1: Search in a mesh term and title and abstract**		
**Concept of interest**		**Settings of interest**
MeSH Term	***Combined with***	***At least one of the following terms in the Title or Abstract***
incivility	("and")	hospital
		operating room
		operating theatre
		Surgery
		intensive care unit
		ICU
		medical team
		physician
		doctor
		nurse
		anesthetist
		anesthesiologist
		anesthesia
		emergency department
		peri-operative
		obstetrics
		gynecology
		CRNA
		pediatrician
		surgeon
		resident
		medical student
		internal medicine
		palliative
		otorhinolaryngology
**Step 2: Search in title and abstract**		
**Concept of interest**		**Settings of interest**
At least one of the following terms in the Title or Abstract	***Combined with ("and")***	***At least one of the following terms in the Title or Abstract***
incivility		hospital
rudeness		operating room
disruptive behavior		operating theatre
unprofessional behavior		Surgery
interpersonal tension		intensive care unit
		ICU
		medical team

Additional Material, Table 1: Search strategy used on Medline

	physician
	doctor
	nurse
	anesthetist
	anesthesiologist
	anesthesia
	emergency department
	peri-operative
	obstetrics
	gynecology
	CRNA
	pediatrician
	surgeon
	resident
	medical student
	internal medicine
	palliative
	otorhinolaryngology
