# Supplementary material for: Predictors and triggers of incivility within healthcare teams: a systematic review of the literature
Source: BMJ Open. 2020 Jun 7;10(6):e035471. doi: 10.1136/bmjopen-2019-035471 (PMC7282335; doi:10.1136/bmjopen-2019-035471)
Supplement: Supplementary data [file bmjopen-2019-035471supp002.pdf]

Additional material, Table 2: details of MERSQI scores

	**design**	**sampling**		**type of data**	**Validity of instrument**			**Data analysis**		**Types of outcome measured**				**Total score**
Author		*Institutions studied*	*Response rate score*		*Internal structure*	*Content*	*Relationship to other variables*	*Appropriateness*	*Complexity*	*Satisfaction, attitudes, perception*	*Knowledge, skills*	*Behaviors*	*Patient/health care outcome*	
Addison and Luparell (2014)	1	1	0.5	1	0	1	0	1	1	1	0	0	0	**7.5**
Alkaabi and Wong (2019)	1	1.5	0.5	1	1	1	1	1	2	1	0	0	0	**11**
Alshehry et al. (2019)	1	1	1.0	1	1	1	1	1	2	1	0	0	0	**11**
Arslan Yürümezoğlu and Kocaman (2019)	1	1	1.0	1	1	1	1	1	2	1	0	0	0	**11**
Bae et al. (2016)	1	1.5	0.5	1	1	1	0	1	2	1	0	0	0	**10**
Bansal (2014)	1	0.5	0.5	1	1	1	0	1	1	1	0	0	0	**8**
Berman-Kishony and Shvarts (2015)	1	0.5	0.5	1	0	1	1	1	2	1	0	0	0	**9**
Birks et al. (2017)	1	1.5	0.5	1	1	1	0	1	2	1	0	0	0	**10**
Bradley et al. (2015)	1	1.5	0.5	1	0	0	0	1	1	1	0	0	0	**7**
Brewer et al. (2013)	1	1.5	1.0	1	1	1	0	1	1	1	0	0	0	**9.5**
Budden et al. (2017)	1	1.5	0.5	1	1	1	0	1	2	1	0	0	0	**10**
Budin et al. (2013)	1	1.5	1.0	1	1	1	1	0	2	1	0	0	0	**10.5**
Chang et al. (2019)	1	1.5	1.5	1	1	1	1	1	2	1	0	0	0	**12**
Elhoseny and Adel (2016)	1	0.5	1.0	1	0	0	0	1	1	1	0	0	0	**6.5**
Elmblad et al. (2014)	1	1.5	0.5	1	1	1	1	1	2	1	0	0	0	**11**

Additional material, Table 2: details of MERSQI scores

Finlayson et al. (2013)	1	1.5	0.5	3	0	1	1	1	2	0	0	2	0	**13**
Goettler et al. (2011)	1	0.5	1.5	1	0	1	0	1	2	1	0	0	0	**9**
Hamblin et al. (2016)	1	1.5	1.5	1	0	1	0	1	2	1	0	0	0	**10**
Heslin et al. (2019)	1	0.5	1.5	3	1	1	1	1	2	1	0	1	0	**14**
Heydari et al. (2015)	1	1.5	1.5	1	1	1	0	0	2	1	0	0	0	**10**
Kaiser (2017)	1	0.5	0.5	1	1	1	1	1	2	1	0	0	0	**10**
Keller et al. (2018)	1	1.5	1.5	1	1	1	1	1	2	1	0	0	0	**12**
Keller et al. (2019)	1	1	1.5	3	1	0	1	1	2	1	0	1	0	**13**
Klingberg et al. (2018)	1	0.5	1.0	1	0	1	1	1	2	1	0	0	0	**9.5**
Layne et al. (2019)	1	0.5	0.5	1	1	1	0	1	2	1	0	0	0	**9**
Lewis and Malecha (2011)	1	1.5	0.5	1	1	1	0	1	2	1	0	0	0	**10**
Minton et al. (2018)	1	1.5	0.5	1	1	1	0	1	2	1	0	0	0	**10**
Mullan et al. (2013)	1	1.5	1.5	1	0	1	0	1	2	1	0	0	0	**10**
Nemeth et al. (2017)	1	0.5	0.5	1	1	1	0	1	2	1	0	0	0	**9**
Rehder et al. (2020)	1	1.5	1.5	1	1	1	1	1	2	1	0	0	0	**12**
Rosenstein and Naylor (2012)	1	1.5	0.5	1	0	1	0	1	1	1	0	0	0	**8**
Rosenstein and O’Daniel (2005)	1	1.5	0.5	1	0	1	0	1	1	1	0	0	0	**8**
Rosenstein and O'Daniel (2008)	1	1.5	0.5	1	0	1	0	1	0	1	0	0	0	**7**

Additional material, Table 2: details of MERSQI scores

Ruvalcaba et al. (2018)	1	1.5	0.5	1	1	1	0	1	2	1	0	0	0	**10**
Sellers et al. (2012)	1	1.5	0.5	1	1	1	0	1	2	1	0	0	0	**10**
Shetty et al. (2016)	1	1.5	0.5	1	0	1	0	1	2	1	0	0	0	**9**
Sliter et al. (2014)	1	1.5	0.5	1	1	1	1	1	2	1	0	0	0	**11**
Small et al. (2015)	1	1.5	0.5	1	0	1	0	1	2	1	0	0	0	**9**
Smith et al. (2018)	1	1.5	0.5	1	1	1	1	1	2	1	0	0	0	**11**
Tikva et al. (2019)	1	1.5	0.5	1	1	0	1	1	2	1	0	0	0	**10**
Veltman (2007)	1	1.5	1.0	1	0	0	0	1	1	1	0	0	0	**7.5**
Villafranca et al. (2019)	1	1.5	0.5	1	1	1	1	1	2	1	0	0	0	**11**
Viotti et al. (2018)	1	1.5	0.5	1	1	1	1	1	2	1	0	0	0	**11**
Walrath et al. (2013)	1	0.5	0.5	1	1	1	0	1	2	1	0	0	0	**9**
