# Supplementary material for: Predictors and triggers of incivility within healthcare teams: a systematic review of the literature
Source: BMJ Open. 2020 Jun 7;10(6):e035471. doi: 10.1136/bmjopen-2019-035471 (PMC7282335; doi:10.1136/bmjopen-2019-035471)
Supplement: Author's manuscript [file bmjopen-2019-035471.draft_revisions.pdf]

BMJ Open

BMJ Open is committed to open peer review. As part of this commitment we make the peer review history of every article we publish publicly available.

When an article is published we post the peer reviewers' comments and the authors' responses online. We also post the versions of the paper that were used during peer review. These are the versions that the peer review comments apply to.

The versions of the paper that follow are the versions that were submitted during the peer review process. They are not the versions of record or the final published versions. They should not be cited or distributed as the published version of this manuscript.

BMJ Open is an open access journal and the full, final, typeset and author-corrected version of record of the manuscript is available on our site with no access controls, subscription charges or pay-per-view fees (http://bmjopen.bmj.com).

If you have any questions on BMJ Open's open peer review process please email info.bmjopen@bmj.com

BMJ Open

Predictors and triggers of incivility within medical teams: A systematic review of the literature

Journal:	*BMJ Open*
Manuscript ID	bmjopen-2019-035471
Article Type:	Original research
Date Submitted by the Author:	02-Nov-2019
Complete List of Authors:	Keller, Sandra; Brigham and Women's Hospital, Center for Surgery and Public Health (CSPH); Harvard Medical School<br>Yule, Steven; Harvard University; STRATUS Center for Medical Simulation<br>Zagarese, Vivian; Virginia Tech<br>Parker, Sarah; Virginia Tech; Fralin Biomedical Research Institute (FBRI) at Virginia Tech Carilion
Keywords:	MEDICAL EDUCATION & TRAINING, Health & safety < HEALTH SERVICES ADMINISTRATION & MANAGEMENT, Quality in health care < HEALTH SERVICES ADMINISTRATION & MANAGEMENT

SCHOLARONE™
Manuscripts

I, the Submitting Author has the right to grant and does grant on behalf of all authors of the Work (as defined in the below author licence), an exclusive licence and/or a non-exclusive licence for contributions from authors who are: i) UK Crown employees; ii) where BMJ has agreed a CC-BY licence shall apply, and/or iii) in accordance with the terms applicable for US Federal Government officers or employees acting as part of their official duties; on a worldwide, perpetual, irrevocable, royalty-free basis to BMJ Publishing Group Ltd ("BMJ") its licensees and where the relevant Journal is co-owned by BMJ to the co-owners of the Journal, to publish the Work in this journal and any other BMJ products and to exploit all rights, as set out in our licence.

The Submitting Author accepts and understands that any supply made under these terms is made by BMJ to the Submitting Author unless you are acting as an employee on behalf of your employer or a postgraduate student of an affiliated institution which is paying any applicable article publishing charge ("APC") for Open Access articles. Where the Submitting Author wishes to make the Work available on an Open Access basis (and intends to pay the relevant APC), the terms of reuse of such Open Access shall be governed by a Creative Commons licence – details of these licences and which Creative Commons licence will apply to this Work are set out in our licence referred to above.

Other than as permitted in any relevant BMJ Author's Self Archiving Policies, I confirm this Work has not been accepted for publication elsewhere, is not being considered for publication elsewhere and does not duplicate material already published. I confirm all authors consent to publication of this Work and authorise the granting of this licence.

For peer review only

Title: Predictors and triggers of incivility within medical teams: A systematic review of the literature

Authors

Sandra Keller[1,2*]

Steven Yule[1,2,3,4]

Vivian Zagarese[5]

Sarah Henrickson Parker[5,6]

[1] Center for Surgery and Public Health, Brigham and Women's Hospital

[2] Harvard Medical School

[3] Department of Surgery, Brigham and Women's Hospital

[4] STRATUS Center for Medical Simulation

[5] Virginia Tech

[6] Fralin Biomedical Research Institute (FBRI) at Virginia Tech Carilion

*Corresponding author:

E-mail: sandrakeller@vtc.vt.edu

Word count: 4175

Abstract

Objective: To explore predictors and triggers of incivility in medical teams

Design: Systematic literature review

Setting: Hospitals delivering non-psychiatric patient care

Participants: Healthcare professionals with different educational backgrounds and working in diverse medical domains

Primary and secondary outcome measures: Predictors and triggers of incivility included personal characteristics of initiators and targets of incivilities, professional backgrounds and domain of professionals involved in uncivil episodes, situational and cultural predictors of incivilities.

Results: Among the 38 studies reviewed, 31 were quantitative and seven qualitative. Initiators of incivility were consistently described as having a difficult personality; yet few studies investigated their other characteristics and motivations. Results were mostly inconsistent regarding individual characteristics of targets of incivilities (e.g. age, gender, ethnicity), despite the high number of studies available. In most studies, participants reported experiencing incivilities mainly within their own professional discipline (e.g. nurse to nurse) rather than across disciplines (e.g. physician to nurse). Further, evidence of specific medical specialties particularly affected by incivility was poor. Surgery was one of the most cited uncivil specialty, with contrasting results based on physicians' ratings of their interactions with surgeons. Finally, situational and cultural predictors of higher incivility levels included high workload, communication or coordination issues, patient safety concerns, lack of support from colleagues and poor leadership in the department. Note that most of the studies assessing situational and cultural aspects relied on cross-sectional surveys assessing participants' perception.

Conclusion: Although a wide range of different predictors and triggers of incivilities are reported in the literature, identifying characteristics of initiators, the and targets of incivilities, have yielded mainly inconsistent results. The use of diverse and high-quality methods is needed to explore the dynamic nature of situational and cultural triggers of incivility. An accurate understanding of these complex dynamics will support the design of efficient interventions to decrease incivility.

For peer review only - http://bmjopen.bmj.com/site/about/guidelines.xhtml

Article summary

Strengths and limitation of the study

- The systematic format of the review allowed us to investigate current empirical findings on predictors of incivility from both medical and nursing literature.
- To explore the predictors and triggers of incivilities, the methods included quantitative and qualitative studies, which allowed an overview of the topic beyond methodological boundaries.
- Examining a wide range of predictors contributes to shed light on which predictors were already extensively investigated and for which predictors more empirical research is needed.
- Quality of the studies included were rather low and the conceptualization of incivility and related terms based mainly on study participants' perception; this is an inherent limitation to the review.

For peer review only - http://bmjopen.bmj.com/site/about/guidelines.xhtml

Introduction

Incivility among healthcare professionals has recently drawn increased attention in the medical world. The potential of incivility to jeopardize optimal patient care – and in turn patient safety, represents one of the major factors that led to their identification as a latent issue in healthcare[1 2]. Defined as behaviors that violate norms of respect but whose intent to harm is ambiguous[3], incivilities are not typically in the scope of legal sanctions despite their negative effects[4].

Healthcare professionals themselves perceive an association between incivilities and decreased patient safety[5]. A medical simulation study found more specifically a negative effect of rude behavior on speaking up in medical students[6]. This result was supported by further simulation studies that also showed a decrease in communication after the expression of incivilities and a poorer performance[7]. In other domains, incivility showed negative effects both on well-being of employees and turnover[8].

Research on the prevalence of the phenomena in different healthcare settings identified that more than three quarters of healthcare employees had already witnessed incivilities by physicians and almost two thirds incivilities by nurses[9]. In another study, 85% of the nurses reported personally having experienced incivilities in the past year[10]. These findings outline the importance of the phenomena and the need for additional efforts in reducing its frequency and impact. However, the design of efficient interventions to reduce incivilities is closely tight to an accurate knowledge of the predictors and triggers of incivility in medical teams. Predictors are not clearly articulated in the literature, and have been explored in a piecemeal fashion.

In the present manuscript, we report the results of a systematic review on predictors of incivility in hospitals, carried out in June 2018. Because a common characteristic of uncivil behaviors is the ambiguity around the intent to harm[3 11], the review investigated closely related and often overlapping terms: incivility, rudeness, disruptive behaviors, interpersonal tensions and the disruptive behavior part of unprofessional behaviors. These concepts describe impolite and rude conduct[12] such as yelling[13], racial or gender bias[14], and also more subtle behaviors such as silences, rebukes[15], gossip and displaced frustration[16]. Also invisibility and carelessness by colleagues can be perceived as incivility[17].

We examined empirical studies that report predictors of incivilities among medical staff in hospitals. We investigated characteristics of both initiators and targets; their professional background, and the situational and cultural predictors of incivilities.

Methods

The search for literature and report of the results were conducted following the PRISMA guidelines[18]. The aim of the literature search was to identify predictors of incivility in medical teams for which research showed empirical evidence. Quantitative and qualitative studies were included.

Eligibility criteria: We included original publications of empirical studies focusing on predictors and triggers of incivilities among healthcare hospital teams. We set no restrictions in terms of year of publication but considered only papers published in English and in peer-reviewed journals.

Information sources and search strategy: We searched journal articles in four different data bases: Medline, CINHAL, PsychInfo and Web of Science in June 2018. The search included incivility related concepts combined with healthcare professions or major services in the hospitals where non-psychiatric patient care takes place. We followed a systematic search and inclusion exclusion criteria (Figure 1). The Medline data base search strategy is included in Additional Material, (Table 1). We hand searched the references for additional articles.

Study records: data management and selection process: Publication records were independently extracted from the databases and transferred into an Endnote File. Duplicate articles were excluded. In a first step, two reviewers (SK and SHP) independently assessed titles and abstracts of the articles for inclusion. All articles potentially reporting empirical original studies on predictors of uncivil behaviors were selected for full text screening. Divergence in coding were resolved by discussion. In a second step, two raters (SK and VZ) screened the full texts to identify studies meeting the inclusion criteria. Again, differences between the two raters were resolved by discussion and the total number of studies selected was 38 (Figure 1).

Risk of bias: We assessed the study quality of quantitative studies with the Medical Education Research Study Quality Instrument (MERSQI) scale. The MERSQI scale is a validated tool originally designed to assess the quality of medical education publications; it is

based on systematic ratings of the study design, sampling, type of data included, validity of measure instruments, data analysis and type of outcome reported[19].

Synthesis:

The main goal of the review was to identify the predictors of incivility reported in empirical studies. We categorized the predictors of incivilities reported in the studies into five categories: (i) individual characteristics of initiators of incivilities, (ii) individual characteristics of targets of incivility, (iii) professional groups involved in incivility episodes, in terms of professional background and medical specialization or hospital department, (iv) situational aspects and (v) cultural determinants. Specific concepts, methods and measurement tools used in the studies were also extracted (***Table 1)***.

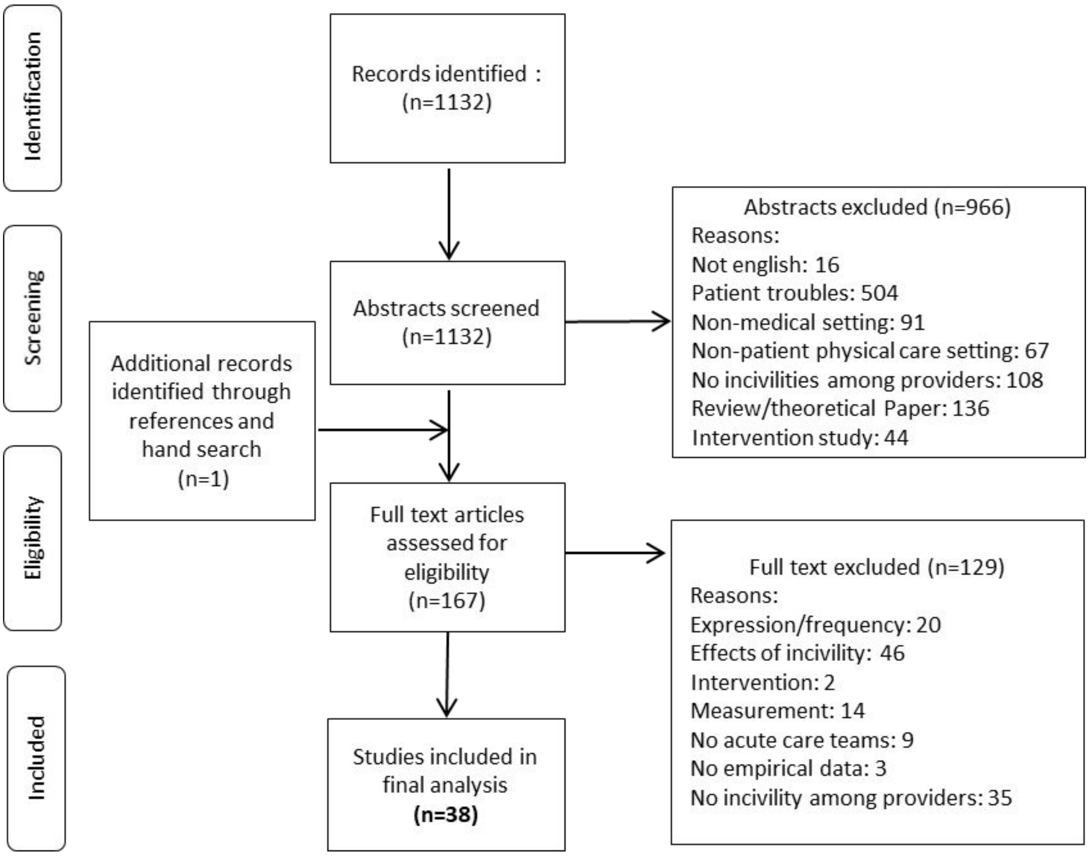

Figure 1. Flow diagram of the selection process of studies included

Results

We will first present descriptive results about the studies, and then discuss their content. Content results are split into initiators, targets, medical specialties, situations, and cultural and organizational characteristics.

Descriptive results of the studies:

Time frame: Studies meeting the inclusion criteria were all published between 2002 and 2018. There was a sharp increase in the number of published studies in 2013, after that the number of published studies remained relatively stable, but on a low frequency level, with four to five published studies per year (***Figure 2***).

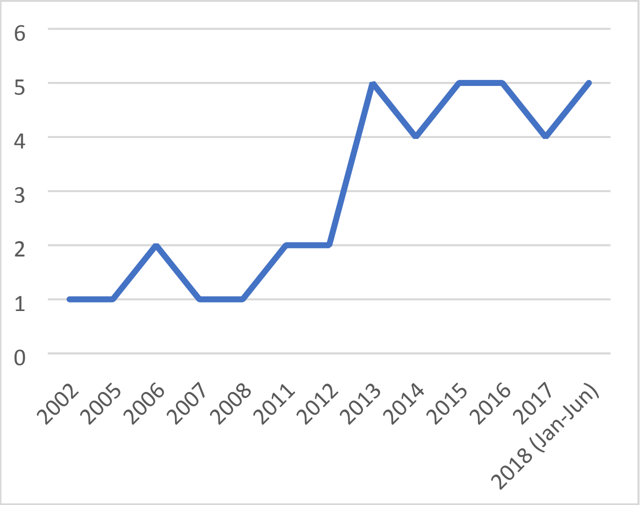

Figure 2. Publication years of the study included (y-axis represents the number of studies)

Methodology of the study included:

Thirty-one of the 38 studies included quantitative analysis and seven were based on a qualitative design (**table 1**).

Among the quantitative studies, the majority, 27 studies, relied on cross-sectional research design and used questionnaires. Other methodologies included analysis of prospective self-reports by the participants (events sampling)[20], data extracted from an institutional electronic reporting systems[21 22] and data collected as part of a physician fitness to practice evaluation program[23].

Qualitative studies included four interview studies[24-27], one observational study[15], one study based on a combination of observations and interviews[28] and one qualitative analysis of error reporting systems[29].

For peer review only - http://bmjopen.bmj.com/site/about/guidelines.xhtml

Quality of studies included:

MERSQI scores, used to assess the quality of the quantitative studies, were relatively low overall, with a mean MERSQI score of 9.5, ranging between 6.5 and 13 on a scale from 5 (lowest possible MERSQI score) to 18 (highest possible MERSQI score) (details of the MERSQI scores for each study are available in *Additional Material, Table 2*). Methodological limitations were often similar across studies. First, many studies relied solely on participants' perceptions, with the exception of one study based on the evaluation of a fitness to practice evaluation committee[23] and an ethnographic observational study[15]. Second, most questionnaire studies reported low response rates, with a response rate below 50% in 21 studies. Third, eight studies described prevalence of disruptive behaviors and their triggers, but did not report more complex statistical analyses.

Predictors of incivility

We present the results for each sub-category of predictors of incivilities, i.e. characteristics of initiators of incivility, characteristics of targets, professional groups and medical domains, situational and cultural predictors (***Table 2***).

Initiators of incivility: When asked about the main triggers of incivilities, medical staff consistently mentioned personality as a major contributor to incivilities or that incivilities were initiated repeatedly by the same individuals[24 26 27 30 31]. One study showed that personality disorders were indeed more frequently diagnosed in physicians evaluated for disruptive behavior than physicians evaluated for other issues (e.g. sexual harassment)[23]. No other study investigated specifically personality characteristics of initiators of incivilities.

Evidence of demographic characteristics of initiators of incivilities was also extremely scarce with one study exploring characteristics of uncivil physicians and two studies exploring the characteristics of uncivil nurses. The only overlapping result across the three studies was that initiators were more likely to be middle-aged or older than their targets[21 23 25]. Two studies found that initiators of incivilities were more likely to belong to the dominant racial group[23 25].

Targets of incivility: Fifteen studies included information on characteristics of medical staff most likely to be targeted by incivilities.

Gender was the most investigated personal characteristic of targets of incivilities. Three studies found that females were more likely than males to be targeted[20 24 32], whereas seven studies found no differences between females and males[13 21 25 33-36]. One study including

nursing students in the UK and Australia, found that females were more likely to report incivilities in the Australian sample whereas in the UK, there was a trend that males were more likely to report incivilities[37].

Age was a further characteristic investigated in association with the experience of incivility. Research on which *age* groups were more likely to be targeted by incivility showed mixed results. Three studies consistently found that younger health professionals were more likely to experience incivilities[10 34 38], whereas four studies did not find differences across age groups[13 14 21 35].

Regarding *professional experience* (a likely correlate with age), two studies showed that less experienced professionals were more likely to be targeted by incivilities[14 36]. However, one study found that more advanced nurse students experienced more incivility in Australia but not in the UK[37] and one study showed no experience effect[33]. There was thus slightly more studies showing that less experienced team members were more often targets of incivility than studies finding contrasting results.

Ethnical background of targets was another characteristic often hypothesized to predict incivilities. Four studies found indeed that healthcare professionals with a non-dominant ethnical background or native language in the country where the study was conducted were more likely to experience incivilities[24 25 36 37], whereas three studies did not find differences across ethnic groups[13 35 38].

Two studies explored the association between experience of incivility and target's *psychological states*. One study found an association between decreased work satisfaction and being targeted by incivilities[35], and another study found similar findings with negative affectivity[13]; yet no association was found with job motivation[35]. It is important to note that these studies were cross-sectional and the association between incivilities and psychological work-related states were not explored over time, which does not allow us to draw causal associations.

Professional background and medical subspecialties:

Results of the studies included allowed exploration of potential differences in the prevalence of incivilities across medical professions and medical domains. We first report differences across professional backgrounds, e.g. nurse and physicians and second, we report comparisons across medical domains (e.g. operating room vs ICU).

For peer review only - http://bmjopen.bmj.com/site/about/guidelines.xhtml

Professional backgrounds: The most often examined research question pertained to the prevalence of incivilities in physicians and nurses, and studies investigated the most likely instigator of incivilities among professional groups. We first present the results of studies that focused on studies conducted with physicians, then studies including mainly nurses and finally diverse professions.

In one study physicians perceived other physicians as the most frequent initiators of incivilities[14] and in another study, physicians perceived incivility by other physicians as incivilities having the most negative impact[39]. Medical interns reported nurses rather than physicians as most frequent initiators of incivilities[14]. In one study, results were less clear, with physicians perceiving about half of the incivilities initiated by nurses and the other half initiated by physicians[40]. Nevertheless, slightly more studies reported that physicians are the primary source of incivilities to other physicians after training completion.

A majority of studies (six) found that nurses perceived other nurses as the most frequent[10 41 42] or most negative source of incivility[39] or that nurses were involved in the majority of incivility incidents reported[21 29]. Note that in one study, the majority of initiators of incivilities towards nurses were described as co-workers or managers, without clear mention of their professional background[10]. Two studies that included nurse students found very similar results [37 38]. Only two studies reported contrasting results, with physicians perceived as the most frequent source of incivilities by nurses[40 43].

Not surprisingly, studies that surveyed diverse medical professionals found mixed results. One study found that physicians were most frequently initiators of incivility[9], whereas another study reported similar rates of incivilities by nurses and physicians[31].

Only three studies focused on the professional groups most likely to be targeted by incivilities. These studies found that nurses[36 40] or scrub technicians, and in general, professions associated with less power in the medical hierarchical system[24] were more frequently targeted by incivilities.

Medical specialties: We addressed the question regarding the extent of the prevalence of incivilities across specific medical specialties. *Surgery* or surgical sub-specialties appeared in four studies as one of the domains with the most incivilities, compared for example to paediatric or ED[12], family or internal medicine doctors[23], the ICU or medical-surgical units[44] and other specialties outside radiology and cardiology[45]. One survey with ICU physicians found contrasting results, showing that surgical specialists were less likely to be uncivil to

For peer review only - http://bmjopen.bmj.com/site/about/guidelines.xhtml

ICU physicians as compared to non-surgical specialists[46]. In the same vein, a study found that interactions with surgeons were not rated more negatively by ED physicians than were interactions with other specialists[20]. Interestingly, in these two latter studies, surgeons were likely to work in other settings than the OR when they interacted with their medical colleagues.

In two studies, *Radiology* appeared to be the specialty associated with the most incivilities. In one study, radiology was followed by general surgery, neurosurgery, cardiology and other specialties[45] and in the other study radiology was compared to medical, surgical and other specialties[20]. One study found contrasting results, with radiology as one of the medical domains with the least incivility, for example compared to surgery, cardiology, trauma and other potentially higher risk specialties[22]. Other medical domains that were associated with more incivilities were *obstetrics*[12 22], long term-care [12], the ED, ICU, cardiology[41 22], whereas a study found that nurses working in the ICU reported the least incivilities compared to other nurses[35].

Interestingly, three studies that included physicians found that incivilities were more likely during collaboration with other departments compared to participants' own department[22 45 46], suggesting that ingroup-outgroup dynamics may also impact incivility. In one of these studies, contradictory results were found for nurses who reported more uncivil behaviors initiated by physicians within their own department than initiated by physicians external to their own departments[22].

Situational influences on incivilities: There is evidence that medical professionals report specific situations as fertile grounds for incivilities. We identified seven different situational triggers investigated in different studies and present these results in **table 2**.

High workload was the most often mentioned trigger of incivilities, reported in nine studies. One questionnaire study did not find an effect of workload, and another study found an effect of workload only in a sample of US nurses but not in a sample of Italian nurses[47]. The second most frequent situational factors identified as trigger of incivilities are related to the non-technical skills of *coordination, communication and teamwork* (e.g. poor communication, lack of teamwork), reported in five different studies. *Patient safety concerns* or poor performance were other factors triggering incivilities reported in three different studies based on ethnographic observations[15], retrospective chart analysis[29] and questionnaires and focus groups[45]. Two studies found that situations in which medical staff who experienced *heavy*

responsibilities may be more prone for incivilities. In two studies conducted in the operating room*, time* management and negotiations were triggers of tense situations[15 28].

Team composition was also investigated as a potential trigger of incivility, with *little familiarity* among team members perceived as enhancing incivilities[24 27]. Finally, *organizational constraints,* defined as factors preventing employees to perform their task efficiently (e.g. because a lack of resources), were perceived as a potential catalyst of incivilities in a cross-sectional survey study[13] and in another study based on incident reports[29].

Some other situational factors each one investigated by a single study and contributing to incivilities in medical teams were fatigue[36], the reason for the interaction, i.e. request for medical investigations[20], compensation or non-work related factors[48]

Culture and organization's characteristics

The relationship of culture, organization of the department, the hospital or of countries to uncivil behavior where investigated by different studies. We included results of studies that did not directly measure culture but closely related concepts, such as the impact of department leaders and studies comparing samples of participants working in different countries.

Leadership was associated with incivilities in several studies. Four studies investigating nurses found that the nurses managers' skills to handle incivilities was a protective factor against incivilities[35 44 45 49]. A study with physician faculty members found similar results, with participants pointing to the lack of reaction of leaders in handling less severe incivilities[27]. Further, transformational leadership was found to be protective of incivilities[12] whereas lack of leadership was associated with increased perceived incivility[36]; none of the study provided data on how transformational leaders contribute to reduced incivility levels. Only one cross-sectional study did not find an association between perceived supervisor support and incivility[13].

Workplace cultures also seem to influence incivilities. For example, three studies found that nurses working in a magnet hospital – a label recognizing the quality of nursing care and the professional development of the nursing workforce[50], were less likely to experience incivilities. Only one study failed to find an effect[13 51]. In three further studies that were conducted with physicians[24 48], respectively with a mixed sample of physicians and nurses[31], the authors found evidence that culture and training contribute to incivilities, suggesting that uncivil behaviors are learnt and fostered during physicians' training. Further, a positive work

For peer review only - http://bmjopen.bmj.com/site/about/guidelines.xhtml

culture and support from colleagues[13 35 49] and a diversity climate, as assessed by the Diversity Climate Scale measuring the perception of the value of diversity by the organization[43] were associated with decreased incivilities in four studies, without evidence of divergent results. In one study, distributive justice, but not procedural justice, was also associated with decreased incivility levels[13].

Few studies focused on the impact of the countries' cultures on incivilities. Two studies, conducted with nurses, included samples from different countries. One found that the prevalence of incivilities was higher in the US compared to the Italian nurse sample. The other study compared Australian with UK nurse students and found that Australian nurse students reported more incivility.

Discussion

This systematic review reports the current state of research related to triggers of uncivil behavior, reporting consistent and inconsistent findings. A first fact is that although the interest for this topic is growing in the medical field, the number of studies reporting empirical work is modest. In addition, the quality scores for most studies, as assessed by MERSQI criteria, was slightly lower than in other samples[19], with only two studies relying on other measurement methods than perceptions of the study participants. An important result of this review is the need for more empirical research of high quality.

Nevertheless, the existing studies cover a wide range of factors that underlie expression of incivility at work. These predictors or triggers of tensions range from the intrinsic characteristics of the people involved in incivility episodes to situational or cultural aspects influencing the emergence of incivilities. Existing models of incivilities in medical teams already include many of the triggers identified empirically, for example the model of triggers of incivilities in the operating room presented by Villafranca, et al. [52] and that describes intrapersonal, organizational and interpersonal factors. However, they are not studied in a systematic way.

Studies investigating initiators of incivilities support an influence of personality on uncivil behavior sometimes described as "bad apples"[24]. However, most of these studies are based on perceptions of study participants. Relatively few studies focused on initiators' perceptions and explored their motivations and interactional context, beyond personality.

For peer review only - http://bmjopen.bmj.com/site/about/guidelines.xhtml

Overall, the review shows that demographics of *targets* are inconsistently related to incivilities. Although explored by fifteen studies, it was not possible to identify consistent gender differences and specific age and ethnic groups as particularly likely targets of incivilities. However, the few studies available on the association between work experience and incivilities show that more experience, often associated with a higher hierarchical status in the organization, is associated with decreased experience of incivilities, indicating that higher task proficiency, but also higher status may be a protective factor. This is in line with the experience of physicians who observed that they were treated with more respect since their promotion to consultant compared to earlier stages of their medical career [45].

In terms of professional background of tension initiators, the dynamics appeared to be more complex than could be expected. Results showed more evidence of incivilities within similar professional groups – sometimes called horizontal violence, compared to inter-professional incivilities. Whereas this result is not surprising for physicians, it shows that nurses, rather than physicians, were in most studies reported as more likely to initiate incivilities. Of note, most studies did not measure nor control for the frequency of interactions within, and between, professional groups; this is an important potential bias. In addition, most studies are based on the perception of a specific professional group which may also be a source of bias [53]. The studies also failed to identify consistent differences among medical specialties, with the exception of surgeons during their work in the OR. This result may be explained by the more stressful work conditions, the closer cooperation and the higher risk tasks performed [22].

Different *situational* aspects influence incivilities in medical teams, with workload, communication and teamwork as most important factors, followed by patient safety issues as compared to other predictors. Among *cultural* factors, leadership and support among the group as well as working in a hospital recognized for excellence in nursing care were among factors recognized as protecting against high incivility levels. Thus, these results suggest that rather than universal professional cultures, local dynamics in specific work situations, departments and hospitals may influence incivilities and should be considered.

Overall, the methodological quality was relatively low for most of the studies reviewed. Methods such as prospective and systematic observation of uncivil interactions[15 20] or relying on hospital surveillance systems[21 29]are rare. Even situational triggers of tensions which need to be studied specifically were investigated with cross-sectional survey studies. However, given the only relatively recent interest in this topic, it is important to note that some of the studies included in the review belong to the very first studies that focused on incivilities in

For peer review only - http://bmjopen.bmj.com/site/about/guidelines.xhtml

medical teams. Thus, methodological weaknesses may be offset by the pioneering character of the work.

Study strengths and limitations of the review

Strengths

One strength of the study was that we included papers based on different methodological approaches to answer the question of the systematic review. This approach allowed to assess similar research questions of studies relying on different methodologies. In addition, this more inclusive approach allows a more extensive overview of the topic.

Because teamwork in medical teams is inherently multidisciplinary, we included research conducted with nurses or a mixed population that was often done in nursing science as well as research conducted with physicians, often initiated by physicians. Further, the search process revealed the impressive number of theoretical or position papers (139) on incivilities compared to empirical studies. The high number of theoretical papers is an indicator for the interest in the topic. However, to understand the phenomenon and what leads to incivilities, there is an urgent need for more empirical research. Only empirical research can inform the conceptualization and the understanding of processes triggering incivilities within medical teams.

Limitations:

A limitation inherent in the topic of incivility is the conceptualizations of incivilities and related behaviors are subjective, because the intent to harm is per definition ambiguous[3]. It is thus important to underline that studies that investigate incivility based on perceptions (i.e. questionnaire studies) cannot claim to measure incivilities and their triggers beyond participants' perceptions.

The few studies focusing on the analysis of specific uncivil events rather than perceptions of those events indicate that uncivil behavior is a complex phenomenon, and much more complex that one initiator behaving in an uncivil way towards a target[15 22]. We did not include conflicts in our search strategy, although conflict behavior can be uncivil. Conflicts are traditionally defined as caused by divergent opinion on the task or process or caused relationship issues and are of longer term[54]. Yet, conflicts situations may well underlie uncivil episodes, and further analyses of conflicts in medical teams may also contribute to the understanding of uncivil episodes in this context[55 56].

Conclusion

Given the known impact of incivilities on both patient care processes[7] and medical professionals' health,[57 58] the need for efficient interventions to reduce incivilities in medical teams is likely to increase. Such interventions need to be based on empirical evidence. The present systematic review showed that most studies investigated general characteristics of initiators and targets of incivilities. Situational aspects that foster incivilities are clearly understudied, so we may underestimate the probability that incivilities are a result of coordination problems. Further studies should concentrate on these situational triggers (cooperation, task requirements). Future incivility research in the medical field also needs to be adopt higher quality methods than current studies. Only if these two conditions are satisfied can empirical results then inform the design of interventions to reduce incivility and the potential harm to providers and patients.

For peer review only - http://bmjopen.bmj.com/site/about/guidelines.xhtml

Table 1. Studies included (n=38): Settings, methods and focus

Authors	Country	Setting	Concept studied	Methods	Participants (N)	Focus	MERSQI score
**Physician to physician**							
Pattani, et al. [27]	Canada	Mixed: Hospitals affiliated with a faculty of medicine	Incivility	Interviews	Faculty members (N=49)	Initiators<br>Situation<br>Culture	n/a[1]
Shetty, et al. [20]	Australia	One emergency department (ED)	Incivility	Prospective self-reports of tone of phone conversations (tool designed by the authors)	Junior and senior physicians rotating or training in the ED (N= 21 physicians, 714 phone consultations)	Target<br>Profession<br>Situation	12
Bradley, et al. [45]	England	Mixed: 3 academic hospitals	Rude, dismissive and aggressive communication	Focus groups and questionnaires (probably designed by the authors)	junior doctors, registrars and consultants (N=606)	Profession<br>Situation<br>Culture	7
**Physicians to all**							
Cochran and Elder [24])	n/a – probably USA	Operating room (OR)	Disruptive behavior	Interviews	Medical students, anesthesiologists, residents, nurses and scrub techs (N=19)	n/a (Open interviews)	n/a
Elhoseny and Adel [48]	Egypt	Medical, surgical, ICU, anesthesia, ED and pathology departments of one hospital	Disruptive behavior	Questionnaire (based on the ACPE and QuantiaMD Survey)	Physicians (N=120)	Situation<br>Culture	6.5
Finlayson, et al. [23]	n/a – probably USA	Mixed: hospitals	Disruptive behavior	Retrospective chart analysis of fitness-for-duty evaluation (Vanderbilt Comprehensive Assessment Program)	Physicians (N=381)	Initiators<br>Profession	13

For peer review only - http://bmjopen.bmj.com/site/about/guidelines.xhtml

Goettler, et al. [22]	USA	Mixed: one academic hospital	Disruptive behavior	Retrospective chart analysis of behaviors reported to the hospital system	Physicians (N=114) for 191 reported events	Initiators Profession	10
Brewer, et al. [34]	USA	Mixed: hospitals (68% of participants), and institutions	Verbal abuse	Questionnaire Verbal abuse scale (by Pejic, 2005), shortened 6-item version	New nurses (up to 6 years as a nurse) (N=1328)	Target Situation Culture	9.5
**All to physicians**							
Mullan, et al. [14]	USA	Mixed: One hospital group	Disruptive behavior	Questionnaire, developed by the authors	Medical interns (394) and attending physicians (40)	Target Profession	10
Klingberg, et al. [46]	Switzerland	ED of one hospital	Incivility, bad manners	Questionnaire, developed by the authors	Physicians (N=50)	Professions	9.5
Lewis and Malecha [44]	USA	OR, medical surgical, ICU, ED and women's services	Workplace incivility	Questionnaire : Nurse Incivility Scale (NIS) (by Guidroz et al., 2007)	Nurses (N=659)	Professions Culture	10
Elmblad, et al. [42]	USA	OR and peri-operative	Workplace incivility	Questionnaires, Nurse Incivility Scale (NIS) (Guidroz, 2010)	Certified registered nurse anesthetist (CRNA) (N=385)	Professions	11
Small, et al. [10]	USA	Probably mixed: Different hospitals	disruptive behaviors and verbal abuse	Questionnaire, developed by the authors	Nurses (N=2821)	Targets Professions	9
Budden, et al. [38]	Australia	Probably mixed	Bullying and harassment	Questionnaire, adapted from a survey designed by Hewett (2010)	Nurses students (N=888)	Target Profession	10
Birks, et al. [37]	Australia and UK	Probably mixed: Nurses recruited via heads of nursing schools	Workplace bullying	Questionnaires, based on the work of Hewett (2010)	Australian (n=883) and UK (n=561) nurses students	Target Profession Culture	10

For peer review only - http://bmjopen.bmj.com/site/about/guidelines.xhtml

**Nurses to nurses**							
Heydari, et al. [33]	Iran	Mixed: Different departments of 3 hospitals	Incivility	Questionnaires: perceived workplace civility climate scale (PWCC)	Nurses (N=157)	Targets Profession	10
Boateng and Adams [25]	Canada	Probably mixed: nurses recruited in 2 cities	Intra-professional conflict	interviews (one-on-one)	Nurses (N=66)	Initiators Targets Situation	n/a
Kaiser [12]	n/a	Mixed: Acute and continuing care (unclear how many facilities included)	Incivility	Questionnaire: Nurses Incivility Scale (NIS) (Guidroz et al., 2010)	Staff nurses (N= 237)	Targets Profession Culture	10
Smith, et al. [49]	USA	Mixed: Medical surgical or critical progressive care units in 5 hospitals	Incivility	Questionnaire: Workplace incivility scale (Cortina et al., 2001)	Nurses (RN) (N =233)	Culture	11
Sellers, et al. [32]	USA	Mixed: 19 facilities	Horizontal violence	Questionnaire: Briles'Sabotage Savvy Quiz	Nurses (N=2659)	Target Culture	10
Keller, et al. [13]	USA	Mixed: Hospitals were the workplace of 75% of participants	Verbal abuse	Questionnaire: Developed by Budin et al. (2013)	Early career nurses (N=1208)	Target Situation Culture	12
Viotti, et al. [47]	USA and Italy	Mixed: one hospital system in USA and one hospital in Italy	Incivility	Questionnaire: co-worker incivility with scale adapted by Sliter et al (2012)	US nurses (n=341) and Italian nurses (n=313)	Situation Culture	11
Budin, et al. [35]	USA	N/a	Verbal abuse	Questionnaires: Shortened version of the Manderino and Banton (1994) Verbal abuse scale (VAS), used by Pejic (2005)	Nurses (N=1407)	Target Profession Situation Culture	10.5
**All incivilities and nurses' point of view**							

For peer review only - http://bmjopen.bmj.com/site/about/guidelines.xhtml

Addison and Luparell [41]	USA	Probably mixed, in 2 rural hospitals	Disruptive behaviors	Questionnaire: developed by Rosenstein & O'Daniel (2005)	57 nurses (N=57)	Professions	7.5
Veltman [59]	USA	Labor and Delivery in 56 hospitals	Disruptive behaviors	Questionnaire: developed by Rosenstein and O'Daniel (2005)	Nurse managers (N=56)	Professions	7.5
Riley and Manias [28]	n/a – probably USA	OR, 3 hospitals	Tension and interpersonal conflicts	ethnographic with observations, group and individual interviews	OR nurses (N=11)	Situations	n/a
McLemore [26]	n/a	n/a	Workplace aggression	Interviews	Nurses (N=4)	Initiators	n/a
Sliter, et al. [43]	USA	n/a	Interpersonal conflict	Questionnaire: Interpersonal conflict at work scale (ICAWS) (Spector and Jex, 1998)	Nurses (N=172)	Profession Culture	11
Nemeth, et al. [60]	USA	Probably mixed, one academic hospital	Lateral violence	Questionnaire, the Lateral Violence in Nursing (LVNS) developed by the authors	Nurses, staff, managers (N=663)	Initiators Situations	9
**All incivilities and all's point of view**							
Rosenstein and Naylor [31]	USA	ED, 20 different EDs	Disruptive behavior	Questionnaire, developed by the authors	Physician, nurses, secretaries or clerks, ED technicians (N=237)	Personality Professions Culture Situations	8
Rosenstein and O'Daniel [9]	USA	Mixed, in 102 hospitals	Disruptive behavior	Questionnaire, developed by the authors	Physicians, nurses, administrative employees and others (N=4530)	Professions	7
Lingard, et al. [15]	n/a	OR in one teaching hospital	Tension	Ethnographic observation	All OR team members (N=n/a)	Situations	n/a

Rosenstein and O'Daniel [40]	USA	Mixed, in 50 hospitals	Disruptive behavior	Questionnaire, designed by the authors	RN, physicians, administrators (N=1509)	Professions	8
Walrath, et al. [39]	USA	Mixed, in one hospital	Disruptive behavior	Questionnaire	RN, MDs, affiliates (N=1559)	Professions	9
Hamblin, et al. [21]	USA	Probably mixed, in a large hospital system with 7 hospitals	Workplace violence	Retrospective chart analysis based on quantitative material	Perpetrators (N=185) for 199 violence incidents	Initiators<br>Targets<br>Professions	11
Hamblin, et al. [29])	USA	Probably mixed, in a large metropolitan hospital system with 7 hospitals	Workplace violence	Retrospective chart analysis based on qualitative material	Violence and incivility incidents for which a catalyst could be identified (N=135)	Professions<br>Situations	n/a
Berman-Kishony and Shvarts [30]	Israel	Probably mixed, one medical center	Disruptive behavior	Questionnaires, developed by the authors based on focus groups and meetings	Nurses (n=76) and physicians (n=58)	Initiators<br>Situations	9
Bae, et al. [36]	USA	Probably mixed, one urban academic medical center	Disruptive behavior	Questionnaires, John Hopkins disruptive behavior survey (JH-DCBS)	Nurses, midwifes, CRNAs, physician assistants, MDs (N=1559)	Targets<br>Professions<br>Situations<br>Culture	10

Note. [1]MERSQI scores are only available for quantitative studies

Table 2. Situational triggers of incivilities in medical teams

Authors	***Situations***	
Brewer, et al. [34]	More physician abuse associated with fewer nurses working than scheduled	*Workload*
Boateng and Adams [25]	If heavy work responsibilities, minority nurses reported conflicts about who did what (expertise)	*Work responsibilities*
Hamblin, et al. [29]	Work behavior: unprofessional behavior, duties and responsibilities, methods of care, poor performance.<br>Work organization: conflicts about tasks and procedures, organizational constraints, interdependence between the workers	*Communication/Teamwork, Patient safety, Work responsibilities Organizational constraints*
Nemeth, et al. [60]	Most highly causal explanation was stress related to inadequate staffing or resources, followed by societal decline in civil behavior	*Workload*
Keller, et al. [13]	Organizational constraints predicted more incivility; no effect of quantitative workload	*Workload (no effect), Organizational constraints*
Pattani, et al. [27]	Infrequent interactions	*Lack of familiarity*
Viotti, et al. [47]	Workload as a predictor of incivility only in the US but not in the Italian sample	*Workload (in one of the study samples)*
Berman-Kishony and Shvarts [30]	High workload is the second most frequent cause reported, followed by poor communication, distrust and disrespect	*Workload, Communication/teamwork*
Budin, et al. [35]	Higher levels of verbal abuse perceived by nurses as associated with: Fewer nurses working than scheduled (staffing shortfalls), less perceived distributive and procedural justice, less promotional opportunities, more organizational constraints, higher quantitative workload	*Workload*
Cochran and Elder [24]	In the operating room, incivility was associated with: unfamiliar teams or trainees, something goes wrong during the operation, when there are differences in opinions with the surgeon while planning the operation	*Familiarity*<br>*Workload or patient safety*

For peer review only - http://bmjopen.bmj.com/site/about/guidelines.xhtml

Rosenstein and Naylor [31]	Delays, inadequate staffing and poor communication were rated less frequently than personality and attitudes	*Workload*<br>*Communication/teamwork*
Riley and Manias [28]	Time: questioning judgement time, controlling speed, estimating surgeon's time, different perceptions of time	*Time*
Elhoseny and Adel [48]	Workload as first root cause (reported by 35%), 15% reported compensation-related factors, Other: non work-related situations (12%)	*Workload*<br>*Non-wok related factors*
Bradley, et al. [45])	Doctors describing the situations in which they are rude: high workload, patient safety compromised, hierarchy	*Workload,*<br>*Patient safety*
Lingard, et al. [15]	Time, resources, roles, safety and sterility, situation control	*Communication/Teamwork,*<br>*Patient safety,*<br>*Time*
Bae, et al. [36]	Triggers of disruptive behaviors at the inter-individual level (e.g. questioning providers about care, lack of teamwork, staff diversity) and intrapersonal level (e.g. lack of competency, fatigue) related to experienced disruptive behaviors.<br>Among nurses only (not physicians) organizational triggers (pressure from high volume, overload, unresolved issues unit culture) were also predictors of disruptive behaviors	*Workload,*<br>*Communication/teamwork*<br>*Patient safety*<br>*Fatigue*
Shetty, et al. [20]	Consultations with requests for investigations	*Request*

Funding statement: This work was sponsored by a grant of the Swiss National Science Foundation, grant number P2NEP1 178574.

Conflict of interests: The authors report no conflict of interest

Authors' contributions:
Study design: Sandra Keller, Sarah Henrickson Parker and Steven Yule
Data analysis: Sandra Keller, Vivian Zagarese and Sarah Henrickson Parker
Drafting the work or critically revising it: Sandra Keller, Steven Yule, Vivian Zagarese, Sarah Henrickson Parker

Acknowledgements: We thank Rita McCandless and Erin M. Smith, Ph.D, for their advices about the literature search process and help in finding the relevant articles to conduct the present literature review. We thank Franziska Tschan, Ph.D for her critical review of the manuscript.

Patient and public involvement statement: It was not appropriate or possible to involve patients or the public in the design, or conduct, or reporting, or dissemination plans of our research

Literature

1. Leape LL, Shore MF, Dienstag JL, et al. Perspective: a culture of respect, part 1: the nature and causes of disrespectful behavior by physicians. *Academic medicine* 2012;87(7):845-52.
2. Flin R. Rudeness at work: British Medical Journal Publishing Group, 2010.
3. Andersson LM, Pearson CM. Tit for tat? The spiraling effect of incivility in the workplace. *Academy of management review* 1999;24(3):452-71.
4. Shapiro J. Confronting unprofessional behaviour in medicine: British Medical Journal Publishing Group, 2018.
5. Rosenstein AH, O'Daniel M. Impact and implications of disruptive behavior in the perioperative arena. *Journal of the American College of Surgeons* 2006;203(1):96-105.
6. Salazar MJB, Minkoff H, Bayya J, et al. Influence of surgeon behavior on trainee willingness to speak up: a randomized controlled trial. *Journal of the American College of Surgeons* 2014;219(5):1001-07.
7. Riskin A, Erez A, Foulk TA, et al. The impact of rudeness on medical team performance: a randomized trial. *Pediatrics* 2015;136(3):487-95.
8. Porath CL, Pearson CM. Emotional and behavioral responses to workplace incivility and the impact of hierarchical status. *Journal of Applied Social Psychology* 2012;42(S1):E326-E57.
9. Rosenstein AH, O'Daniel M. A survey of the impact of disruptive behaviors and communication defects on patient safety. *The Joint Commission Journal on Quality and Patient Safety* 2008;34(8):464-71.
10. Small CR, Porterfield S, Gordon G. Disruptive behavior within the workplace. *Applied Nursing Research* 2015;28(2):67-71. doi: 10.1016/j.apnr.2014.12.002
11. Hershcovis MS, Barling J. Towards a multi-foci approach to workplace aggression: A meta-analytic review of outcomes from different perpetrators. *Journal of Organizational Behavior* 2010;31(1):24-44.
12. Kaiser JA. The relationship between leadership style and nurse-to-nurse incivility: turning the lens inward. *J Nurs Manag* 2017;25(2):110-18. doi: 10.1111/jonm.12447
13. Keller R, Krainovich-Miller B, Budin W, et al. Predictors of nurses' experience of verbal abuse by nurse colleagues. *Nurs Outlook* 2018;66(2):190-203. doi: 10.1016/j.outlook.2017.10.006 [published Online First: 2018/04/25]
14. Mullan CP, Shapiro J, McMahon GT. Interns' experiences of disruptive behavior in an academic medical center. *Journal of graduate medical education* 2013;5(1):25-30. doi: 10.4300/jgme-d-12-00025.1 [published Online First: 2014/01/10]
15. Lingard L, Reznick R, Espin S, et al. Team communications in the operating room: talk patterns, sites of tension, and implications for novices. *Academic Medicine* 2002;77(3):232-37.
16. Oja KJ. Incivility and Professional Comportment in Critical Care Nurses. *AACN advanced critical care* 2017;28(4):345-50.
17. Abdollahzadeh F, Asghari E, Doshmangir L, et al. Workplace Incivility as an Extensively Used, But Seldom Defined Concept in Nursing. *Nurs Midwifery Stud* 2017;6(2):e41029.
18. Moher D, Shamseer L, Clarke M, et al. Preferred reporting items for systematic review and meta-analysis protocols (PRISMA-P) 2015 statement. *Systematic reviews* 2015;4(1):1.
19. Reed DA, Cook DA, Beckman TJ, et al. Association between funding and quality of published medical education research. *Jama* 2007;298(9):1002-09.
20. Shetty AL, Vaghasiya M, Boddy R, et al. Perceived incivility during emergency department phone consultations. *Emerg Med Australas* 2016;28(3):256-61. doi: 10.1111/1742-6723.12564
21. Hamblin LE, Essenmacher L, Ager J, et al. Worker-to-Worker Violence in Hospitals Perpetrator Characteristics and Common Dyads. *Workplace Health Saf* 2016;64(2):51-56. doi: 10.1177/2165079915608856

For peer review only - http://bmjopen.bmj.com/site/about/guidelines.xhtml

22. Goettler CE, Butler TS, Shackleford P, et al. Physician Behavior: Not Ready for 'Never'land. *The American Surgeon* 2011;77(12):1600-05.
23. Finlayson AJR, Dietrich MS, Neufeld R, et al. Restoring professionalism: the physician fitness-for-duty evaluation. *Gen Hosp Psych* 2013;35(6):659-63. doi: 10.1016/j.genhosppsych.2013.06.009
24. Cochran A, Elder WB. A model of disruptive surgeon behavior in the perioperative environment. *Journal of the American College of Surgeons* 2014;219(3):390-98.
25. Boateng GO, Adams TL. "Drop dead ... I need your job": An exploratory study of intra-professional conflict amongst nurses in two Ontario cities. *Soc Sci Med* 2016;155:35-42. doi: 10.1016/j.socscimed.2016.02.045
26. McLemore MR. CJON reporter. Workplace aggression: beginning a dialogue. *Clinical Journal of Oncology Nursing* 2006;10(4):455-56. doi: 10.1188/06.CJON.455-456
27. Pattani R, Ginsburg S, Johnson AM, et al. Organizational factors contributing to incivility at an academic medical center and systems-based solutions: a qualitative study. *Academic Medicine* 2018;93(10):1569.
28. Riley R, Manias E. Governing time in operating rooms. *Journal of clinical nursing* 2006;15(5):546-53.
29. Hamblin LE, Essenmacher L, Upfal MJ, et al. Catalysts of worker-to-worker violence and incivility in hospitals. *Journal of Clinical Nursing* 2015;24(17/18):2458-67. doi: 10.1111/jocn.12825
30. Berman-Kishony T, Shvarts S. Universal versus tailored solutions for alleviating disruptive behavior in hospitals. *Isr J Health Policy Res* 2015;4:12. doi: 10.1186/s13584-015-0018-7
31. Rosenstein AH, Naylor B. Incidence and impact of physician and nurse disruptive behaviors in the emergency department. *The Journal of emergency medicine* 2012;43(1):139-48.
32. Sellers KF, Millenbach L, Ward K, et al. The Degree of Horizontal Violence in RNs Practicing in New York State. *J Nurs Adm* 2012;42(10):483-87. doi: 10.1097/NNA.0b013e31826a208f
33. Heydari A, Rad M, Rad M. Evaluating the Incivility between Staff Nurses and Matrons Employed in Iran. *Acta Fac Medicae Naiss* 2015;32(2):137-46. doi: 10.1515/afmnai-2015-0014
34. Brewer CS, Kovner CT, Obeidat RF, et al. Positive work environments of early-career registered nurses and the correlation with physician verbal abuse. *Nurs Outlook* 2013;61(6):408-16. doi: 10.1016/j.outlook.2013.01.004
35. Budin WC, Brewer CS, Chao YY, et al. Verbal Abuse From Nurse Colleagues and Work Environment of Early Career Registered Nurses. *J Nurs Scholarsh* 2013;45(3):308-16. doi: 10.1111/jnu.12033
36. Bae SH, Dang D, Karlowicz KA, et al. Triggers Contributing to Health Care Clinicians' Disruptive Behaviors. *Journal of patient safety* 2016 doi: 10.1097/pts.0000000000000288 [published Online First: 2016/11/05]
37. Birks M, Cant RP, Budden LM, et al. Uncovering degrees of workplace bullying: A comparison of baccalaureate nursing students' experiences during clinical placement in Australia and the UK. *Nurse Educ Pract* 2017;25:14-21. doi: 10.1016/j.nepr.2017.04.011
38. Budden LM, Birks M, Cant R, et al. Australian nursing students' experience of bullying and/or harassment during clinical placement. *Collegian* 2017;24(2):125-33. doi: 10.1016/j.colegn.2015.11.004
39. Walrath JM, Dang D, Nyberg D. An Organizational Assessment of Disruptive Clinician Behavior Findings and Implications. *J Nurs Care Qual* 2013;28(2):110-21. doi: 10.1097/NCQ.0b013e318270d2ba
40. Rosenstein AH, O'Daniel M. Original Research: Disruptive Behavior and Clinical Outcomes: Perceptions of Nurses and Physicians: Nurses, physicians, and administrators say that clinicians' disruptive behavior has negative effects on clinical outcomes. *AJN The American Journal of Nursing* 2005;105(1):54-64.
41. Addison K, Luparell S. Rural Nurses' Perception of Disruptive Behaviors and Clinical Outcomes: A Pilot Study. *Online Journal of Rural Nursing & Health Care* 2014;14(1):66-82. doi: 10.14574/ojrnhc.v14i1.300

For peer review only - http://bmjopen.bmj.com/site/about/guidelines.xhtml

42. Elmblad R, Kodjebacheva G, Lebeck L. Workplace Incivility Affecting CRNAs: A Study of Prevalence, Severity, and Consequences With Proposed Interventions. *AANA Journal* 2014;82(6):437-45.
43. Sliter M, Boyd E, Sinclair R, et al. Inching Toward Inclusiveness: Diversity Climate, Interpersonal Conflict and Well-Being in Women Nurses. *Sex Roles* 2014;71(1-2):43-54. doi: 10.1007/s11199-013-0337-5
44. Lewis PS, Malecha A. The impact of workplace incivility on the work environment, manager skill, and productivity. *J Nurs Adm* 2011;41(1):41-47.
45. Bradley V, Liddle S, Shaw R, et al. Sticks and stones: investigating rude, dismissive and aggressive communication between doctors. *Clin Med* 2015;15(6):541-45. doi: 10.7861/clinmedicine.15-6-541
46. Klingberg K, Gadelhak K, Jegerlehner SN, et al. Bad manners in the Emergency Department: Incivility among doctors. *PLoS One* 2018;13(3):11. doi: 10.1371/journal.pone.0194933
47. Viotti S, Converso D, Hamblin LE, et al. Organisational efficiency and co-worker incivility: A cross-national study of nurses in the USA and Italy. *J Nurs Manag* 2018 doi: 10.1111/jonm.12587 [published Online First: 2018/01/11]
48. Elhoseny TA, Adel A. Disruptive physician behaviors and their impact on patient care in a health insurance hospital in Alexandria, Egypt. *Journal of the Egyptian Public Health Association* 2016;91(2):80-85.
49. Smith JG, Morin KH, Lake ET. Association of the nurse work environment with nurse incivility in hospitals. *J Nurs Manag* 2018;26(2):219-26. doi: 10.1111/jonm.12537
50. Kramer M, Schmalenberg C. Magnet hospitals: Part I. Institutions of excellence. *The Journal of nursing administration* 1988;18(1):13-24.
51. Association AN. ANCC Magnet Recognition Program® [Available from: https://www.nursingworld.org/organizational-programs/magnet/ accessed November 1st 2019.
52. Villafranca A, Hamlin C, Enns S, et al. Disruptive behaviour in the perioperative setting: a contemporary review. *Canadian Journal of Anesthesia/Journal canadien d'anesthésie* 2017;64(2):128-40.
53. Müller P, Tschan F, Keller S, et al. Assessing Perceptions of Teamwork Quality Among Perioperative Team Members. *AORN journal* 2018;108(3):251-62.
54. Jehn KA. A qualitative analysis of conflict types and dimensions in organizational groups. *Administrative science quarterly* 1997:530-57.
55. Bochatay N, Bajwa NM, Cullati S, et al. A multilevel analysis of professional conflicts in health care teams: insight for future training. *Academic Medicine* 2017;92(11S):S84-S92.
56. Katz JD. Conflict and its resolution in the operating room. *Journal of clinical anesthesia* 2007;19(2):152-58.
57. Wing T, Regan S, Spence Laschinger HK. The influence of empowerment and incivility on the mental health of new graduate nurses. *J Nurs Manag* 2015;23(5):632-43.
58. Babenko-Mould Y, Laschinger HK. Effects of incivility in clinical practice settings on nursing student burnout. *International journal of nursing education scholarship* 2014;11(1):145-54.
59. Veltman LL. Disruptive behavior in obstetrics: a hidden threat to patient safety. *American Journal of Obstetrics and Gynecology* 2007;196(6):587. e1-87. e5.
60. Nemeth LS, Stanley KM, Martin MM, et al. Lateral Violence in Nursing Survey: Instrument Development and Validation. *Healthcare* 2017;5(3):12. doi: 10.3390/healthcare5030033

Table 1 Additional Material: Search strategy used on Medline

**Step 1: Search in a mesh term and title and abstract**		
**Concept of interest**		**Settings of interest**
MeSH Term	***Combined with***	***At least one of the following terms in the Title or Abstract***
incivility	("and")	hospital
		operating room
		operating theatre
		Surgery
		intensive care unit
		ICU
		medical team
		physician
		doctor
		nurse
		anesthetist
		anesthesiologist
		anesthesia
		emergency department
		peri-operative
		obstetrics
		gynecology
		CRNA
		pediatrician
		surgeon
		resident
		medical student
		internal medicine
		palliative
		otorhinolaryngology
**Step 2: Search in title and abstract**		
**Concept of interest**		**Settings of interest**
At least one of the following terms in the Title or Abstract	***Combined with ("and")***	***At least one of the following terms in the Title or Abstract***
incivility		hospital
rudeness		operating room
disruptive behavior		operating theatre
unprofessional behavior		Surgery
interpersonal tension		intensive care unit
		ICU
		medical team

For peer review only

Table 1 Additional Material: Search strategy used on Medline

physician
doctor
nurse
anesthetist
anesthesiologist
anesthesia
emergency department
peri-operative
obstetrics
gynecology
CRNA
pediatrician
surgeon
resident
medical student
internal medicine
palliative
otorhinolaryngology

For peer review only

Additional material, Table 2: details of MERSQI scores

	**design**	**sampling**		**type of data**	**Validity of instrument**			**Data analysis**		**Types of outcome measured**				**Total score**
Author		*Institutions studied*	*Response rate score*		*Internal structure*	*Content*	*Relationship to other variables*	*Appropriateness*	*Complexity*	*Satisfaction, attitudes, perception*	*Knowledge, skills*	*Behaviors*	*Patient/health care outcome*	
Addison and Luparell (2014)	1	1	0.5	1	0	1	0	1	1	1	0	0	0	**7.5**
Bae et al. (2016)	1	1.5	0.5	1	1	1	0	1	2	1	0	0	0	**10**
Berman-Kishony and Shvarts (2015)	1	0.5	0.5	1	0	1	1	1	2	1	0	0	0	**9**
Birks et al. (2017)	1	1.5	0.5	1	1	1	0	1	2	1	0	0	0	**10**
Budden et al. (2017)	1	1.5	0.5	1	1	1	0	1	2	1	0	0	0	**10**
Bradley et al. (2015)	1	1.5	0.5	1	0	0	0	1	1	1	0	0	0	**7**
Brewer et al. (2013)	1	1.5	1.0	1	1	1	0	1	1	1	0	0	0	**9.5**
Budin et al. (2013)	1	1.5	1.0	1	1	1	1	0	2	1	0	0	0	**10.5**
Elhoseny and Adel (2016)	1	0.5	1.0	1	0	0	0	1	1	1	0	0	0	**6.5**
Elmblad et al. (2014)	1	1.5	0.5	1	1	1	1	1	2	1	0	0	0	**11**
Finlayson et al. (2013)	1	1.5	0.5	3	0	1	1	1	2	0	0	2	0	**13**
C. E. Goettler et al. (2011)	1	0.5	1.5	1	0	1	0	1	2	1	0	0	0	**9**
Hamblin et al. (2016)	1	1.5	1.5	1	0	1	0	1	2	1	0	0	0	**10**
Heydari et al. (2015)	1	1.5	1.5	1	1	1	0	0	2	1	0	0	0	**10**
Kaiser (2017)	1	0.5	0.5	1	1	1	1	1	2	1	0	0	0	**10**

For peer review only

Additional material, Table 2: details of MERSQI scores

Keller et al. (2018)	1	1.5	1.5	1	1	1	1	1	2	1	0	0	0	**12**
Klingberg et al. (2018)	1	0.5	1.0	1	0	1	1	1	2	1	0	0	0	**9.5**
Lewis and Malecha (2011)	1	1.5	0.5	1	1	1	0	1	2	1	0	0	0	**10**
Mullan et al. (2013)	1	1.5	1.5	1	0	1	0	1	2	1	0	0	0	**10**
Nemeth et al. (2017)	1	0.5	0.5	1	1	1	0	1	2	1	0	0	0	**9**
Rosenstein and Naylor (2012)	1	1.5	0.5	1	0	1	0	1	1	1	0	0	0	**8**
Rosenstein and O'Daniel (2005)	1	1.5	0.5	1	0	1	0	1	1	1	0	0	0	**8**
Rosenstein and O'Daniel (2008)	1	1.5	0.5	1	0	1	0	1	0	1	0	0	0	**7**
Sellers et al. (2012)	1	1.5	0.5	1	1	1	0	1	2	1	0	0	0	**10**
Shetty et al. (2016)	1	1.5	0.5	1	0	1	0	1	2	1	0	0	0	**9**
Small et al. (2015)	1	1.5	0.5	1	0	1	0	1	2	1	0	0	0	**9**
JSmith et al. (2018)	1	1.5	0.5	1	1	1	1	1	2	1	0	0	0	**11**
Sliter et al. (2014)	1	1.5	0.5	1	1	1	1	1	2	1	0	0	0	**11**
Veltman (2007)	1	1.5	1.0	1	0	0	0	1	1	1	0	0	0	**7.5**
Viotti et al. (2018)	1	1.5	0.5	1	1	1	1	1	2	1	0	0	0	**11**
Walrath et al. (2013)	1	0.5	0.5	1	1	1	0	1	2	1	0	0	0	**9**

For peer review only

PRISMA 2009 Checklist

Section/topic	#	Checklist item	Reported on page #
**TITLE**			
Title	1	Identify the report as a systematic review, meta-analysis, or both.	1
**ABSTRACT**			
Structured summary	2	Provide a structured summary including, as applicable: background; objectives; data sources; study eligibility criteria, participants, and interventions; study appraisal and synthesis methods; results; limitations; conclusions and implications of key findings; systematic review registration number.	2
**INTRODUCTION**			
Rationale	3	Describe the rationale for the review in the context of what is already known.	4
Objectives	4	Provide an explicit statement of questions being addressed with reference to participants, interventions, comparisons, outcomes, and study design (PICOS).	4
**METHODS**			
Protocol and registration	5	Indicate if a review protocol exists, if and where it can be accessed (e.g., Web address), and, if available, provide registration information including registration number.	
Eligibility criteria	6	Specify study characteristics (e.g., PICOS, length of follow-up) and report characteristics (e.g., years considered, language, publication status) used as criteria for eligibility, giving rationale.	5
Information sources	7	Describe all information sources (e.g., databases with dates of coverage, contact with study authors to identify additional studies) in the search and date last searched.	5
Search	8	Present full electronic search strategy for at least one database, including any limits used, such that it could be repeated.	See additional Material Table 1
Study selection	9	State the process for selecting studies (i.e., screening, eligibility, included in systematic review, and, if applicable, included in the meta-analysis).	5-6
Data collection process	10	Describe method of data extraction from reports (e.g., piloted forms, independently, in duplicate) and any processes for obtaining and confirming data from investigators.	5
Data items	11	List and define all variables for which data were sought (e.g., PICOS, funding sources) and any assumptions and simplifications made.	
Risk of bias in individual studies	12	Describe methods used for assessing risk of bias of individual studies (including specification of whether this was done at the study or outcome level), and how this information is to be used in any data synthesis.	5
Summary measures	13	State the principal summary measures (e.g., risk ratio, difference in means).	6

For peer review only

For peer review only - http://bmjopen.bmj.com/site/about/guidelines.xhtml

PRISMA 2009 Checklist

Synthesis of results	14	Describe the methods of handling data and combining results of studies, if done, including measures of consistency (e.g., I^2) for each meta-analysis.	6

Section/topic	#	Checklist item	Reported on page #
Risk of bias across studies	15	Specify any assessment of risk of bias that may affect the cumulative evidence (e.g., publication bias, selective reporting within studies).	
Additional analyses	16	Describe methods of additional analyses (e.g., sensitivity or subgroup analyses, meta-regression), if done, indicating which were pre-specified.	
**RESULTS**			
Study selection	17	Give numbers of studies screened, assessed for eligibility, and included in the review, with reasons for exclusions at each stage, ideally with a flow diagram.	6
Study characteristics	18	For each study, present characteristics for which data were extracted (e.g., study size, PICOS, follow-up period) and provide the citations.	17
Risk of bias within studies	19	Present data on risk of bias of each study and, if available, any outcome level assessment (see item 12).	Additional Material Table 2
Results of individual studies	20	For all outcomes considered (benefits or harms), present, for each study: (a) simple summary data for each intervention group (b) effect estimates and confidence intervals, ideally with a forest plot.	
Synthesis of results	21	Present results of each meta-analysis done, including confidence intervals and measures of consistency.	
Risk of bias across studies	22	Present results of any assessment of risk of bias across studies (see Item 15).	Additional Material Table 2
Additional analysis	23	Give results of additional analyses, if done (e.g., sensitivity or subgroup analyses, meta-regression [see Item 16]).	
**DISCUSSION**			
Summary of evidence	24	Summarize the main findings including the strength of evidence for each main outcome; consider their relevance to key groups (e.g., healthcare providers, users, and policy makers).	8-13
Limitations	25	Discuss limitations at study and outcome level (e.g., risk of bias), and at review-level (e.g., incomplete retrieval of identified research, reporting bias).	15
Conclusions	26	Provide a general interpretation of the results in the context of other evidence, and implications for future research.	16
**FUNDING**			

For peer review only - http://bmjopen.bmj.com/site/about/guidelines.xhtml

PRISMA 2009 Checklist

Funding	27	Describe sources of funding for the systematic review and other support (e.g., supply of data); role of funders for the systematic review.	

From: Moher D, Liberati A, Tetzlaff J, Altman DG, The PRISMA Group (2009). Preferred Reporting Items for Systematic Reviews and Meta-Analyses: The PRISMA Statement. PLoS Med 6(6): e1000097. doi:10.1371/journal.pmed1000097

For more information, visit: **www.prisma-statement.org**.

For peer review only

For peer review only - http://bmjopen.bmj.com/site/about/guidelines.xhtml

BMJ Open

Predictors and triggers of incivility within healthcare teams: A systematic review of the literature

Journal:	*BMJ Open*
Manuscript ID	bmjopen-2019-035471.R1
Article Type:	Original research
Date Submitted by the Author:	05-Mar-2020
Complete List of Authors:	Keller, Sandra; Brigham and Women's Hospital, Center for Surgery and Public Health (CSPH); Harvard Medical School<br>Yule, Steven; Harvard University; STRATUS Center for Medical Simulation<br>Zagarese, Vivian; Virginia Tech<br>Parker, Sarah; Virginia Tech; Fralin Biomedical Research Institute (FBRI) at Virginia Tech Carilion
**Primary Subject Heading**:	Medical education and training
Secondary Subject Heading:	Communication, Medical education and training
Keywords:	MEDICAL EDUCATION & TRAINING, Health & safety < HEALTH SERVICES ADMINISTRATION & MANAGEMENT, Quality in health care < HEALTH SERVICES ADMINISTRATION & MANAGEMENT

SCHOLARONE™
Manuscripts

I, the Submitting Author has the right to grant and does grant on behalf of all authors of the Work (as defined in the below author licence), an exclusive licence and/or a non-exclusive licence for contributions from authors who are: i) UK Crown employees; ii) where BMJ has agreed a CC-BY licence shall apply, and/or iii) in accordance with the terms applicable for US Federal Government officers or employees acting as part of their official duties; on a worldwide, perpetual, irrevocable, royalty-free basis to BMJ Publishing Group Ltd ("BMJ") its licensees and where the relevant Journal is co-owned by BMJ to the co-owners of the Journal, to publish the Work in this journal and any other BMJ products and to exploit all rights, as set out in our licence.

The Submitting Author accepts and understands that any supply made under these terms is made by BMJ to the Submitting Author unless you are acting as an employee on behalf of your employer or a postgraduate student of an affiliated institution which is paying any applicable article publishing charge ("APC") for Open Access articles. Where the Submitting Author wishes to make the Work available on an Open Access basis (and intends to pay the relevant APC), the terms of reuse of such Open Access shall be governed by a Creative Commons licence – details of these licences and which Creative Commons licence will apply to this Work are set out in our licence referred to above.

Other than as permitted in any relevant BMJ Author's Self Archiving Policies, I confirm this Work has not been accepted for publication elsewhere, is not being considered for publication elsewhere and does not duplicate material already published. I confirm all authors consent to publication of this Work and authorise the granting of this licence.

For peer review only

Title: Predictors and triggers of incivility within healthcare teams: A systematic review of the literature

Authors

Sandra Keller[1,2*]

Steven Yule[1,2,3,4]

Vivian Zagarese[5]

Sarah E. Parker[5,6]

[1] Center for Surgery and Public Health, Brigham and Women's Hospital

[2] Harvard Medical School

[3] Department of Surgery, Brigham and Women's Hospital

[4] STRATUS Center for Medical Simulation

[5] Virginia Tech

[6] Fralin Biomedical Research Institute (FBRI) at Virginia Tech Carilion

*Corresponding author:

E-mail: sandrakeller@vtc.vt.edu

Word count: 4175

For peer review only - http://bmjopen.bmj.com/site/about/guidelines.xhtml

Abstract

Objectives: To explore predictors and triggers of incivility in medical teams, defined as behaviors that violate norms of respect but whose intent to harm is ambiguous.

Design: Systematic literature review of quantitative and qualitative empirical studies.

Data Sources: Database searches according to the PRISMA guideline in Medline, CINHAL, PsychInfo, Web of Science, and Embase up to January 2020.

Eligibility Criteria: Original empirical quantitative and qualitative studies focusing on predictors and triggers of incivilities in hospital healthcare teams, excluding psychiatric care.

Data extraction and synthesis: Of the 1397 publications screened, 53 were included (44 quantitative and 9 qualitative studies); publication date ranged from 2002 to January 2020.

Results: Based on the MERSQI scores, the quality of the quantitative studies were medium (mean MERSQI score of 9.93), but quality of studies increased with publication year ($r=.52$; $P<0.001$). Initiators of incivility were consistently described as having a difficult personality; yet few studies investigated their other characteristics and motivations. Results were mostly inconsistent regarding individual characteristics of targets of incivilities (e.g. age, gender, ethnicity); but less experienced healthcare professionals were more exposed to incivility. In most studies, participants reported experiencing incivilities mainly within their own professional discipline (e.g. nurse to nurse) rather than across disciplines (e.g. physician to nurse). Evidence of specific medical specialties particularly affected by incivility was poor, with surgery as one of the most cited uncivil specialties. Finally, situational and cultural predictors of higher incivility levels included high workload, communication or coordination issues, patient safety concerns, lack of support and poor leadership.

Conclusions: Although a wide range of predictors and triggers of incivilities are reported in the literature, identifying characteristics of initiators and the targets of incivilities yielded inconsistent results. The use of diverse and high-quality methods is needed to explore the dynamic nature of situational and cultural triggers of incivility.

Article summary

Strengths and limitation of the study

- To our knowledge, this is the first systematic review on current empirical findings identifying predictors of incivility from both medical and nursing literature.

- To explore the predictors and triggers of incivilities, methods included quantitative and qualitative studies, which allowed an overview of the topic beyond methodological boundaries.
- Examining a wide range of predictors contributes to shed light on which predictors were already extensively investigated and for which predictors more empirical research is needed.
- Overall, the quality of the included studies was low and the conceptualization of incivility and related terms based mainly on retrospective studies of study participants' perception; this is an inherent limitation to the review.

For peer review only - http://bmjopen.bmj.com/site/about/guidelines.xhtml

Introduction

Incivility among healthcare professionals has recently drawn increased attention in the medical world. The potential of incivility to jeopardize optimal patient care – and in turn patient safety, represents one of the major factors that led to their identification as a latent issue in healthcare[1 2]. Defined as behaviors that violate norms of respect but whose intent to harm is ambiguous[3], incivilities are not typically in the scope of legal sanctions - despite their negative effects[4].

Healthcare professionals themselves perceive an association between incivilities and decreased patient safety[5]. For example, a simulation study found a negative effect of rude behavior on speaking up in medical students[6]. This result was supported by other simulation studies showing a decrease in communication after the expression of incivilities and also showing negative impact on performance[7]. In other domains, incivility showed negative effects both on well-being of employees and turnover[8].

More than three quarters of healthcare employees have witnessed incivilities by physicians and almost two thirds incivilities by nurses[9]. In another study, 85% of the nurses reported having personally experienced incivilities in the past year[10]. These findings outline the importance and prevalence of the phenomena and the need for additional efforts to reduce frequency and impact. The design of efficient interventions to reduce incivilities is closely tied to an accurate knowledge of the *predictors and triggers* of incivility in health teams. Predictors are not clearly articulated in the literature and have been explored in a piecemeal fashion. This literature review aims to provide a broad overview of the current empirical knowledge on predictors of incivility.

In this manuscript, we report the results of a systematic review on predictors of incivility in hospitals, including papers up to January 2020. Because a common characteristic of uncivil behaviors is the ambiguity around the intent to harm[3 11], the review investigated closely related and often overlapping terms: incivility, rudeness, disruptive behaviors, interpersonal tensions and the disruptive behavior part of unprofessional behaviors. These concepts describe impolite and rude conduct[12] and include overt behaviors such as yelling[13], and racial or gender bias[14]. It also includes more subtle behaviors such as silences, rebukes[15], gossip and displaced frustration[16]. Treating others like they are invisible or carelessness by colleagues can also be perceived as incivility[17].

The medical, and in particular, the nursing literature also uses other terms such as verbal abuse (e.g. accusing, blaming, yelling, insulting, humiliating, swearing[13]), horizontal or lateral violence (i.e. violence across members of a same professional group) and bullying, a longer-term form of lateral violence[18] to describe episodes of incivility or violence among health professionals. Because the mechanisms underlying more severe or longer term intra-personal conflictual behaviors may differ from the ones underlying incivility, we restricted the focus of the present literature review on incivilities and low intensity aggressive behaviors.

We examined empirical studies that report predictors of incivilities among healthcare teams in hospitals, including physicians, nursing and other professionals involved in patient care in hospitals. We investigated characteristics of both initiators and targets, their professional background, and the situational and cultural predictors of incivilities.

Methods

The search for literature and the reporting of the results were conducted following the PRISMA guidelines[19]. Quantitative and qualitative studies were included.

Eligibility criteria: We included original publications of empirical studies focusing on predictors and triggers of incivilities among healthcare hospital teams. Studies conducted with medical or nursing students were included if they focused on clinical experiences of the students. Studies conducted in classroom educational settings were considered as not relevant because we aimed at capturing the dynamics of incivility in the clinical and patient care settings, where time pressure and stress are potentially higher. We included studies related to healthcare professionals working mainly in hospitals, with the exception of psychiatric hospitals. This decision was motivated by the potentially higher prevalence of patient incivility in psychiatric care settings whereas the focus of this reviews is on incivility within healthcare teams. We set no restrictions in terms of year of publication and searched the full data bases up to January 2020, but considered only papers published in English and in peer-reviewed journals with empirical findings related to predictors for incivilities.

Information sources and search strategy: One author (SK) searched publications in four different data bases: Medline, CINHAL, PsychInfo, Web of Science and Embase in January 2020. The search included incivility related concepts combined with healthcare professions or major services in the hospitals where non-psychiatric patient care takes place. We followed a systematic search and inclusion exclusion criteria (Figure 1). The Medline data base search

For peer review only - http://bmjopen.bmj.com/site/about/guidelines.xhtml

strategy is included in Additional Material (Table 1). We hand searched the references for additional articles.

Study records: data management and selection process: Publication records were independently extracted from the databases and transferred into an Endnote File. Duplicate articles were excluded. Publication records were then transferred from Endnote to a spread sheet before coding. A multiple-choice menu was created to code the reasons of exclusion. In a first step, two reviewers (SK and SHP) independently assessed titles and abstracts of the articles for inclusion. All articles potentially reporting empirical original studies on predictors of uncivil behaviors were selected for full text screening. Divergence in coding were resolved by discussion. In a second step, two raters (SK and VZ) screened the full texts to identify studies meeting the inclusion criteria. Again, differences between the two raters were resolved by discussion within the rating team (SK, SHP, VZ). See Figure 1 for a schema of the data management process.

Risk of bias: The quality of quantitative studies was assessed with the Medical Education Research Study Quality Instrument (MERSQI) scale by one author (SK). The MERSQI scale is a validated tool originally designed to assess the quality of medical education publications; it is based on systematic ratings of the study design, sampling, type of data included, validity of measure instruments, data analysis and type of outcome reported[20].

Synthesis:

The main goal of the review was to identify the predictors of incivility reported in empirical studies. We categorized the predictors of incivilities reported in the studies into five categories: (i) individual characteristics of initiators of incivilities, (ii) individual characteristics of targets of incivility, (iii) professional groups involved in incivility episodes, in terms of professional background and medical specialization or hospital department, (iv) situational aspects and (v) cultural determinants. Specific concepts, methods and measurement tools used in the studies were also extracted (***Table 1)***.

[insert figure 1 here]

Figure 1. Flow diagram of the selection process of studies included

Patient and public involvement:

It was not appropriate or possible to involve patients or the public in the design, or conduct, or reporting, or dissemination plans of our research.

For peer review only - http://bmjopen.bmj.com/site/about/guidelines.xhtml

Results

The total number of studies selected was 53. We first present descriptive results about the studies, and then discuss their content. Content results are split into initiators, targets, medical specialties, situations, and cultural and organizational characteristics.

Descriptive results of the studies:

Time frame: Studies meeting the inclusion criteria were published between 2002 and 2020. There was a sharp increase in the number of published studies in 2013, after that the number of published studies remained relatively stable, but on a low frequency level, with four to five published studies per year; since 2018, the number of studies again increased.

Methodology of the included studies:

Forty-four of the 53 studies included quantitative analysis and nine were based on a qualitative design (**Table 1**).

Among the quantitative studies, the majority, 39 studies, relied on cross-sectional research design and used questionnaires. Other methodologies included analysis of prospective self-reports by the participants (events sampling)[21], data extracted from or collected in partly with an institutional electronic reporting systems[22 23 24],data collected as part of a physician fitness to practice evaluation program[25], or direct observations[26].

Qualitative studies included four interview studies[27-30], one observational study[15], one study based on a combination of observations and interviews[31] and one qualitative analysis of reporting systems[32].

Quality of studies included:

MERSQI scores, used to assess the quality of the quantitative studies, were relatively low overall, with a mean MERSQI score of 9.93, ranging between 6.5 and 14 on a scale from 5 (lowest possible MERSQI score) to 18 (highest possible MERSQI score) (details of the MERSQI scores for each study are available in *Additional Material, Table 2*). More recent publications showed higher MERSQI scores; we found a correlation of .52 ($p < .001$) between year of publication an MERSQI scores, see Figure 2).

For peer review only - http://bmjopen.bmj.com/site/about/guidelines.xhtml

Methodological limitations were often similar across studies. First, many studies relied solely on participants' perceptions, with the exception of four studies based on the evaluation of a fitness to practice evaluation committee[25], an expert committee examining the perspectives of multiple professionals involved in a same incivility event[24], systematic observations[26] and an ethnographic observational study[15]. Second, most questionnaire studies reported low response rates, with a response rate below 50% in 28 studies. Third, nine studies described prevalence of disruptive behaviors and their triggers, but did not report more complex statistical analyses.

[Insert Figure 2 here]

Figure 2. Scatter plot and trend line of year of publication and MERSQI scores of the quantitative studies meeting the inclusion criteria of the current review

Predictors of incivility

The results for each sub-category of predictors of incivilities and the situational and cultural predictors are summarized in Table 2.

Initiators of incivility: When asked about the main triggers of incivilities, healthcare professionals consistently mentioned personality as a major contributor to incivilities or that incivilities were initiated repeatedly by the same individuals[27 29 30 33-36]. One study showed that personality disorders were indeed more frequently diagnosed in physicians evaluated for disruptive behavior than physicians evaluated for other issues (e.g. sexual harassment)[25]. No other study investigated specific personality characteristics of initiators of incivilities.

Evidence of demographic characteristics of initiators of incivilities was scarce, with one study exploring characteristics of uncivil physicians and two studies exploring the characteristics of uncivil nurses. The only overlapping result across the three studies was that initiators were more likely to be middle-aged or older than their targets[22 25 28]. Two studies found that initiators of incivilities were more likely to belong to the dominant racial group[25 28]. Physicians initiating incivility were predominantly males[23 25 35].

Targets of incivility: Fifteen studies included information on characteristics of healthcare professionals most likely to be targeted by incivilities. In Figure 3, we present an overview of the empirical evidence.

Gender was the most investigated personal characteristic of targets of incivilities. Six studies conducted with health care professionals with different professional backgrounds found that females were more likely than males to be targeted[21 27 37-40]. Eight studies, also including different professional backgrounds, found no differences between females and males[13 22 28 41-45]. One study including nursing students in the UK and Australia, found that females were more likely to report incivilities in the Australian sample whereas in the UK, there was a trend that males were more likely to report incivilities[46].

Research on which *age* groups were more likely to be targeted by incivility showed mixed results. Five studies found that younger health professionals were more likely to experience incivilities[10 39 42 47 48], whereas four studies did not find differences across age groups[13 14 22 43]. Among nursing students, one study showed that older nursing students reported more incivility[40], and another study found that nurses aged 25-27, but not aged 22-24, experienced more incivility than older nurses[45].

Regarding *professional experience* (which is likely correlated with age), six studies showed that less experienced professionals were more likely to be targeted by incivilities[14 38 39 44 45 49]. Among nursing students, there was some evidence that advanced nursing students were more exposed to incivility[40 46]. One study showed no experience effect[41]. Overall, studies showed that less experienced team members were more often targets of incivility, but that different dynamics may operate during nursing education.

Ethnical background of targets was another characteristic often hypothesized to predict incivilities. Five studies found indeed that healthcare professionals with a non-dominant ethnical background or non-native speakers in the country where the study was conducted were more likely to experience incivilities[27 28 44 46 48], whereas four studies did not find differences across ethnic groups[13 39 43 47]. Of note, two studies found contrasting results with non-native speakers reporting less incivility[40 48]; yet in one these studies, non-native speakers were also unsure about identifying the concept of incivility[48].

Few studies focused on nurses' educational background[10 13 38 41 44] (e.g. diploma vs baccalaureate[38]), shift type[13 42] or job tenure[22 44]. Cross-sectional studies investigating the

association between psychological states such as work satisfaction and incivility are scarce and do not allow to identify consistent results [13 43].

[Insert Figure 3 here]

Figure 3. Strength of current empirical evidence on the association between characteristics of healthcare professionals and exposure to incivility

Professional background and medical subspecialties:

Results of the studies included allowed exploration of potential differences in the prevalence of incivilities across medical professions and medical domains. We first report differences across professional backgrounds, e.g. nurse and physicians and second, we report comparisons across medical domains (e.g. operating room vs ICU).

Professional backgrounds: The most often examined research question pertained to the prevalence of incivilities in physicians and nurses, and studies investigated the most likely instigator of incivilities among professional groups.

Perception of physicians: In one study physicians perceived other physicians as the most frequent initiators of incivilities[14] and in another study, physicians perceived incivility by other physicians as incivilities having the most negative impact[50]. Medical interns reported nurses rather than physicians as most frequent initiators of incivilities[14]. In one study, results were less clear, with physicians perceiving about half of the incivilities initiated by nurses and the other half initiated by physicians[51]. Nevertheless, slightly more studies reported that physicians are the primary source of incivilities to other physicians after training completion.

Perception of nurses. A majority of studies (seven) found that nurses perceived other nurses as the most frequent or most negative source of incivility[10 50 52 53], three studies were conducted with nursing students[46-48]. Four studies reported contrasting results, with physicians perceived as the most frequent source of incivilities by nurses[38 51 54] or nursing managers[55].

Studies including professionals from a variety of backgrounds. Not surprisingly, studies that surveyed diverse medical professionals found mixed results. One study found that physicians were most frequently initiators of incivility[9], whereas another study reported similar rates of incivilities by nurses and physicians[34]. Two studies based on institutional reports found that

For peer review only - http://bmjopen.bmj.com/site/about/guidelines.xhtml

nurses were more often involved in incivility episodes compared to other professions[22]. Of note, one of these studies did not include most incivility episodes reported by physicians[22]. Three operation room (OR) studies showed contrasting results, with attending surgeons more likely than the other OR healthcare professionals to initiate uncivil episodes[24 26 36].

Five studies focused on the professional groups most likely to be targeted by incivilities. These studies found that nurses or scrub technicians [26 39 44 51], and in general, professions associated with less power in the medical hierarchical system[27] – more junior surgeons in one study[26] - were more frequently targeted by incivilities.

Medical specialties: We addressed the question regarding the prevalence of incivilities across specific medical specialties. *Surgery* or surgical sub-specialties appeared in five studies as one of the domains with the most incivilities, compared for example to paediatric or emergency departments [12], family or internal medicine doctors[25], the intensive care units (ICU) or medical-surgical units[56] and other specialties outside radiology and cardiology[49], with professionals spending more time in the OR reporting higher incivility levels[39]. One survey with ICU physicians found contrasting results, showing that surgical specialists were less likely to be uncivil to ICU physicians as compared to non-surgical specialists[57]. In the same vein, a study found that interactions with surgeons were rated by emergency department (ED) physicians similarly as interactions with other specialists[21]. Interestingly, in these two latter studies, surgeons were likely to work in other settings than the OR when they interacted with their medical colleagues.

In two studies, *radiology* appeared to be the specialty associated with the most incivilities. In one study, radiology was followed by general surgery, neurosurgery, cardiology and other specialties[49] and in the other study radiology was compared to medical, surgical and other specialties[21]. One study found contrasting results, with radiology as one of the medical domains with the least incivility, for example compared to surgery, cardiology, trauma and other potentially higher risk specialties[23]. Other medical domains that were associated with more incivilities were *obstetrics*[12 23] – with one study showing contrasting results[38], long term-care [12], the ED, ICU, cardiology[52 23], whereas a study found that nurses working in the ICU reported the least incivilities compared to other nurses[43]. However, two studies did not find different perceived incivility levels when comparing general, intermediate and ICU, specialty care and nursing clinical support[58], respectively general ward, ICU, emergency room, and operating room[45].

For peer review only - http://bmjopen.bmj.com/site/about/guidelines.xhtml

Three studies that included physicians found that incivilities were more likely during *collaboration with other departments* compared to participants' own department[23 49 57], suggesting that intergroup dynamics may also impact incivility. In one of these studies, contradictory results were found for nurses who reported more uncivil behaviors initiated by physicians within their own department than initiated by physicians external to their own departments[23].

Situational influences on incivilities: There is evidence that medical professionals report specific situations as fertile grounds for incivilities. We identified seven different situational triggers investigated in different studies and present these results in **Table 2**.

High workload was the most often mentioned trigger of incivilities, reported in ten studies. One questionnaire study did not find an effect of workload, and another study found an effect of workload only in a sample of US nurses but not in a sample of Italian nurses[59]. The second most frequent situational factors identified as trigger of incivilities are related to the non-technical skills of *coordination, communication and teamwork* (e.g. poor communication, lack of teamwork), reported in nine different studies. *Patient safety concerns* or poor performance were other factors triggering incivilities reported in three different studies based on ethnographic observations[15], retrospective chart analysis[32] and questionnaires and focus groups[49]. Two studies found that situations in which healthcare professionals who experienced *heavy responsibilities* may be more prone for incivilities. In two studies conducted in the operating room, *time* management and negotiations were triggers of tense situations[15 31].

Team composition was also investigated as a potential trigger of incivility, with *little familiarity* among team members perceived as enhancing incivilities[27 30]. Finally, *organizational constraints,* defined as factors preventing employees to perform their task efficiently (e.g. because a lack of resources), were perceived as a potential catalyst of incivilities [13 32 36], as were task difficulties and stress[26 36].

Some other situational factors investigated by a single study and contributing to incivilities in healthcare teams were fatigue[44], personality conflicts[24], the reason for the interaction, i.e. request for medical investigations[21], compensation or non-work related factors[60].

Culture and organization's characteristics

For peer review only - http://bmjopen.bmj.com/site/about/guidelines.xhtml

The relationship of culture, organization of the department, the hospital or of countries to uncivil behavior where investigated by different studies. We included results of studies that did not directly measure culture but closely related concepts, such as the impact of department leaders and studies comparing samples of participants working in different countries.

Leadership was associated with incivilities in several studies. Four studies investigating nurses found that the nurses managers' skills to handle incivilities [43 49 56 61] or setting the right tone[62] was a protective factor against incivilities. A study with physician faculty members found similar results, with participants pointing to the lack of reaction of leaders in handling less severe incivilities[30]. Further, transformational [12] or authentic [63] leadership were found to be protective of incivilities whereas lack of leadership was associated with increased perceived incivility[44]; none of the studies provided data on how transformational leaders contribute to reduced incivility levels. Only one cross-sectional study did not find an association between perceived supervisor support and incivility[13].

Workplace culture also seems to influence incivilities. For example, three studies found that nurses working in a magnet hospital – a label recognizing the quality of nursing care and the professional development of the nursing workforce[64], were less likely to experience incivilities. Only one study failed to find an effect[13 65] and one study found an association between incivility and private founded hospitals[39]. In three further studies that were conducted with physicians[27 60], respectively with a mixed sample of physicians and nurses[34], the authors found evidence that culture and training contribute to incivilities, suggesting that uncivil behaviors are learned and fostered during physicians' training. Further, a positive work culture and support from colleagues or the organization [13 43 61 66-68] and a diversity climate[54] were associated with decreased incivilities in seven studies, without evidence of divergent results. In one study, distributive justice, but not procedural justice, was also associated with decreased incivility levels[13].

Few studies focused on the impact of the countries' cultures on incivilities. Two studies, conducted with nurses, included samples from different countries. One found that the prevalence of incivilities was higher in the US compared to the Italian nurse sample. The other study compared Australian with UK nurse students and found that Australian nurse students reported more incivility.

For peer review only - http://bmjopen.bmj.com/site/about/guidelines.xhtml

Discussion

This systematic review reports the current state of research related to triggers of uncivil behavior, reporting consistent and inconsistent findings. Although the interest for this topic has been present for in the past years in the medical field, the number of studies reporting empirical work only recently started to increase. In addition, the quality scores for most studies, as assessed by MERSQI criteria, were comparable to other samples[20], with only three quantitative studies and one qualitative study relying on other measurement methods than perceptions of the study participants. An important result of this review is the need for more empirical research of high quality.

Nevertheless, the existing studies cover a wide range of factors that underlie expression of incivility at work. These predictors or triggers of tensions range from the intrinsic characteristics of the people involved in incivility episodes to situational or cultural aspects influencing the emergence of incivilities. Existing models of incivilities in healthcare teams already include many of the triggers identified empirically, for example the model of triggers of incivilities in the operating room presented by Villafranca, et al. [69] that describes intrapersonal, organizational and interpersonal factors. However, they are not studied in a systematic way.

Studies investigating initiators of incivilities support an influence of personality on uncivil behavior sometimes described as “bad apples”[27]. However, most of these studies are based on perceptions of study participants. Relatively few studies focused on initiators’ perceptions and explored their motivations and interactional context, beyond personality.

Overall, the review shows that demographics of *targets* are not consistently related to incivilities. Although explored by fifteen studies, it was not possible to identify consistent gender differences and specific age and ethnic groups as particularly likely targets of incivilities. However, the studies available on the association between work experience and incivilities show that more experience, often associated with a higher hierarchical status in the organization, is associated with decreased experience of incivilities. This indicates that higher task proficiency, and higher status, may be protective factors. This finding is in line with the experience of physicians who observed that they were treated with more respect after their promotion to consultant compared to earlier stages of their medical career [49].

In terms of professional background of tension initiators, the dynamics appeared to be more complex than could be expected. Results showed more evidence of incivilities within similar

For peer review only - http://bmjopen.bmj.com/site/about/guidelines.xhtml

professional groups, as compared to inter-professional incivilities. Whereas this result is not surprising for physicians, it shows that nurses, rather than physicians, were in most studies reported as more likely to initiate incivilities. Of note, most studies did not measure nor control for the frequency of interactions within, and between, professional groups; this is an important potential bias. In addition, most studies are based on the perception of a specific professional group which may also be a source of bias [70]. The studies also failed to identify consistent differences among medical specialties, with the exception of surgeons during their work in the OR. This result may be explained by the more stressful work conditions, the closer cooperation and the higher risk tasks performed [23].

Different *situational* aspects influence incivilities in healthcare teams, with workload, communication and teamwork as most important factors, followed by patient safety issues as compared to other predictors. Among *cultural* factors, leadership and support among the group as well as working in a hospital recognized for excellence in nursing care were among factors recognized as protecting against high incivility levels. Thus, these results suggest that rather than universal professional cultures, local dynamics in specific work situations, departments and hospitals may influence incivilities and should be considered.

Overall, the methodological quality was relatively low for many of the studies reviewed. Methods such as prospective and systematic observation of uncivil interactions[15 21 26] or relying on hospital surveillance systems[22 24 32]are rare. Even situational triggers of tensions which need to be studied specifically were investigated with cross-sectional survey studies. However, given the only relatively recent interest in this topic, it is important to note that some of the studies included in the review belong to the very first studies that focused on incivilities in healthcare teams. Thus, methodological weaknesses may be offset by the pioneering character of the work, and more recently published papers showed better methodological quality.

Study strengths and limitations of the review

Strengths

One strength of the study was that we included papers based on different methodological approaches to answer the question of the systematic review. This approach allowed to assess similar research questions of studies relying on different methodologies. In addition, this more inclusive approach allows a more extensive overview of the topic.

For peer review only - http://bmjopen.bmj.com/site/about/guidelines.xhtml

Because teamwork in healthcare teams is inherently multidisciplinary, we included research conducted with nurses or a mixed population that was often done in nursing science as well as research conducted with physicians, often initiated by physicians. Further, the search process revealed the impressive number of theoretical or position papers (183) on incivilities much more than empirical studies. The high number of theoretical papers is an indicator for the interest in the topic. To understand the phenomenon and what leads to incivilities, there is an urgent need for more empirical research, and in particular research that goes beyond questionnaire studies. Only empirical research can inform the conceptualization and the understanding of processes triggering incivilities within healthcare teams.

Limitations:

A limitation inherent in the topic of incivility is the conceptualizations of incivilities and related behaviors are subjective, because the intent to harm is per definition ambiguous[3]. It is thus important to underline that studies that investigate incivility based on perceptions (i.e. questionnaire studies) cannot claim to measure incivilities and their triggers beyond participants' perceptions. However, recent studies are promising, showing that perceived incivility can be efficiently assessed with validated tools (see Harris and colleagues for a review[71]) and methods relying on systematic analysis of institutional reports[24] or observations[26] are emerging.

The few studies focusing on the analysis of specific uncivil events rather than perceptions of those events indicate that uncivil behavior is a complex phenomenon, and much more complex that one initiator behaving in an uncivil way towards a target[15 23]. We did not include conflicts in our search strategy, although conflict behavior can be uncivil. Conflicts are traditionally defined as caused by divergent opinion on the task or process or caused relationship issues and are of longer term[72]. Yet, conflicts situations may well underlie uncivil episodes, and further analyses of conflicts in healthcare teams may also contribute to the understanding of uncivil episodes in this context[73 74]. Similarly, studies that included terms such as horizontal violence, lateral violence, bullying, or other forms of aggression without reference to one of our search terms were not included. This allowed to focus the review specifically on less severe forms of rudeness. Yet, there is currently a lack of consistency on the definition of terms related to rude behaviors in the literature [18 75]. We thus cannot exclude that our search strategy did not allow to capture studies that relied on terms usually describing intentional intent to harm (e.g. aggression[75]) and whose definitions widely overlapped with incivility in individual works.

For peer review only - http://bmjopen.bmj.com/site/about/guidelines.xhtml

Conclusion

Given the known impact of incivilities on both patient care processes[7] and healthcare professionals' health,[76 77] the need for efficient interventions to reduce incivilities in healthcare teams is likely to increase. Such interventions need to be based on empirical evidence. The present systematic review showed that most studies investigated general characteristics of initiators and targets of incivilities. Situational aspects that foster incivilities are clearly understudied, so we may underestimate the probability that incivilities are a result of coordination problems. Further studies should concentrate on these situational triggers (cooperation, task requirements). Future incivility research in the medical field also needs to be adopt higher quality methods than current studies. Only if these two conditions are satisfied can empirical results then inform the design of interventions to reduce incivility and the potential harm to providers and patients. Interventions at the organizational level are particularly likely to benefit from this research since healthcare organizations can influence to a certain degree the design of work processes, leadership within departments and cultural aspects that tackle rather than promote incivility.

For peer review only - http://bmjopen.bmj.com/site/about/guidelines.xhtml

Table 1. Studies included (n=35): Settings, methods and predictors included (highlighted yellow are the NEW studies FOUND JANUARY 2020). Here I copy pasted the table from the main document submitted and just added the lines for the new papers and let the other ones unchanged
Table 1. Studies included (n=38): Settings, methods and focus

Authors	year	Country	Setting	Concept studied	Methods	Participants (N)	Focus	MERSQI score
**Physician to physician**								
Pattani, et al. [30]	2018	Canada	Mixed: Hospitals affiliated with a faculty of medicine	Incivility	Interviews	Faculty members (N=49)	Initiators<br>Situation<br>Culture	n/a[1]
Shetty, et al. [21]	2016	Australia	One emergency department (ED)	Incivility	Prospective self-reports of tone of phone conversations (tool designed by the authors)	Junior and senior physicians rotating or training in the ED (N= 21 physicians, 714 phone consultations)	Target<br>Profession<br>Situation	12
Bradley, et al. [49]	2015	England	Mixed: 3 academic hospitals	Rude, dismissive and aggressive communication	Focus groups and questionnaires (probably designed by the authors)	junior doctors, registrars and consultants (N=606)	Profession<br>Situation<br>Culture	7
**Physicians to all**								
Elhoseny and Adel [60]	2016	Egypt	Medical, surgical, ICU, anesthesia, ED and pathology departments of one hospital	Disruptive behavior	Questionnaire (based on the ACPE and QuantiaMD Survey)	Physicians (N=120)	Situation<br>Culture	6.5
Bansal [78]	2014	Na	One tertiary care hospital	Disruptive behaviors	Questionnaire, developed by the authors	Doctors, nurses and technicians (N = 614)	Initiators	8
Cochran and Elder [27]	2014	n/a – probably USA	Operating room (OR)	Disruptive behavior	Interviews	Medical students, anesthesiologists, residents, nurses and scrub techs (N=19)	n/a (Open interviews)	n/a
Brewer, et al. [42]	2013	USA	Mixed: hospitals (68% of participants), and	Verbal abuse	Questionnaire Verbal abuse scale (by Pejic, 2005),	New nurses (up to 6 years as a nurse)	Target<br>Situation	9.5

For peer review only - http://bmjopen.bmj.com/site/about/guidelines.xhtml

			institutions		shortened 6-item version	(N=1328)	Culture	
Finlayson, et al. [25]	2013	n/a – probably USA	Mixed: hospitals	Disruptive behavior	Retrospective chart analysis of fitness-for-duty evaluation (Vanderbilt Comprehensive Assessment Program)	Physicians (N=381)	Initiators Profession	13
Goettler, et al. [23]	2011	USA	Mixed: one academic hospital	Disruptive behavior	Retrospective chart analysis of behaviors reported to the hospital system	Physicians (N=114) for 191 reported events	Initiators Profession	10
**All to physicians**								
Klingberg, et al. [57]	2018	Switzerland	ED of one hospital	Incivility, bad manners	Questionnaire, developed by the authors	Physicians (N=50)	Professions	9.5
Birks, et al. [46]	2017	Australia and UK	Probably mixed: Nurses recruited via heads of nursing schools	Workplace bullying	Questionnaires, based on the work of Hewett (2010)	Australian (n=883) and UK (n=561) nurses students	Target Profession Culture	10
Budden, et al. [47]	2017	Australia	Probably mixed	Bullying and harassment	Questionnaire, adapted from a survey designed by Hewett (2010)	Nurses students (N=888)	Target Profession	10
Small, et al. [10]	2015	USA	Probably mixed: Different hospitals	disruptive behaviors and verbal abuse	Questionnaire, developed by the authors	Nurses (N=2821)	Targets Professions	9
Elmblad, et al. [53]	2014	USA	OR and peri-operative	Workplace incivility	Questionnaires, Nurse Incivility Scale (NIS) (Guidroz, 2010)	Certified registered nurse anesthetist (CRNA) (N=385)	Professions	11
Mullan, et al. [14]	2013	USA	Mixed: One hospital group	Disruptive behavior	Questionnaire, developed by the authors	Medical interns (394) and attending physicians (40)	Target Profession	10
Lewis and Malecha [56]	2011	USA	OR, medical surgical, ICU, ED and women's services	Workplace incivility	Questionnaire : Nurse Incivility Scale (NIS) (by Guidroz et al., 2007)	Nurses (N=659)	Professions Culture	10
**Nurses to nurses**								

Alkaabi and Wong [63]	2019	Canada	Mixed, probably many different hospitals	Incivility	SIS (Straightforward incivility scale) by Leiter and Day (2013), only the manager part	New graduate nurses (N=1020)		11
Arslan Yürümezoğlu and Kocaman [66]	2019	Turkey	Mixed: in 2 state academic/teaching hospitals	Incivility	Workplace Incivility Scale developed by Cortina, Magley, Williams, and Langhout (2001)	Nurses (N=574)	Culture	11
Jones, et al. [79]	2019	South Korea	Mixed; 3 tertiary hospitals	Verbal abuse	Verbal Abuse Questionnaire (Pejic, 2005).	Nurses (N=378)	Targets Profession Culture	12
Tikva, et al. [67]	2019	Israel	Probably mixed, many different hospitals	Disruptive behavior	Questionnaire developed by the authors	Nurses (N=567)	Culture	10
Keller, et al. [13]	2018	USA	Mixed: Hospitals were the workplace of 75% of participants	Verbal abuse	Questionnaire: Developed by Budin et al. (2013)	Early career nurses (N=1208)	Target Situation Culture	12
Smith, et al. [61]	2018	USA	Mixed: Medical surgical or critical progressive care units in 5 hospitals	Incivility	Questionnaire: Workplace incivility scale (Cortina et al., 2001)	Nurses (RN) (N =233)	Culture	11
Viotti, et al. [59]	2018	USA and Italy	Mixed: one hospital system in USA and one hospital in Italy	Incivility	Questionnaire: co-worker incivility with scale adapted by Sliter et al (2012)	US nurses (n=341) and Italian nurses (n=313)	Situation Culture	11
Kaiser [12]	2017	n/a	Mixed: Acute and continuing care (unclear how many facilities included)	Incivility	Questionnaire: Nurses Incivility Scale (NIS) (Guidroz et al., 2010)	Staff nurses (N= 237)	Targets Profession Culture	10
Boateng and Adams [28]	2016	Canada	Probably mixed: nurses recruited in 2 cities	Intra-professional conflict	interviews (one-on-one)	Nurses (N=66)	Initiators Targets Situation	n/a
Budin, et al. [43]	2013	USA	N/a	Verbal abuse	Questionnaires: Shortened	Nurses (N=1407)	Target	10.5

					version of the Manderino and Banton (1994) Verbal abuse scale (VAS), used by Pejic (2005)		Profession Situation Culture	
Sellers, et al. [37]	2012	USA	Mixed: 19 facilities	Horizontal violence	Questionnaire: Briles'Sabotage Savvy Quiz	Nurses (N=2659)	Target Culture	10
**All incivilities and nurses' point of view**								
Alshehry, et al. [38]	2019	Saudi Arabia	Mixed, 2 government hospitals	Incivility	Nursing Incivility Scale (NIS) developed by Guidroz, Burnfield-Geimer, Clark, Schwetschenau, and Jex (2010)	Nurses (N=378)	Targets Professions	11
Layne, et al. [58]	2019	USA	One hospital, level 1 trauma center	Incivility	Nurse Incivility Scale (NIS) (Guidroz et al., 2010)	Nurses (N=414)	Professions	9
Minton and Birks [62]	2019	NZ	Mixed, different hospitals	Bullying/Harrassment	Questionnaire, Student Experience of Bullying During Clinical Placement (SEBDCP) survey, by Budden et al., 2017	Nursing students enrolled in a bachelor program (N=296)	Culture	10
Minton, et al. [48]	2018	New Zealand	Probably mixed, hospitals and other settings	Bullying/Harassment	Questionnaire, Student Experience of Bullying During Clinical Placement (SEBDCP) survey, by Budden et al., 2017	Nursing students enrolled in a bachelor program (N=296)	Targets Profession	9.5
Ruvalcaba, et al. [40]	2018	USA	Probably mixed, in diverse hospitals	Incivility	Questionnaire, Uncivil Clinical Behavior in Nursing Education (UBCNE) tool (Anthony et al., 2014)	Nursing students (N=975)	Targets	10
Nemeth, et al. [80]	2017	USA	Probably mixed, one academic hospital	Lateral violence	Questionnaire, the Lateral Violence in Nursing (LVNS) developed by the authors	Nurses, staff, managers (N=663)	Initiators Situations	9

For peer review only - http://bmjopen.bmj.com/site/about/guidelines.xhtml

Addison and Luparell [52]	2014	USA	Probably mixed, in 2 rural hospitals	Disruptive behaviors	Questionnaire, developed by Rosenstein & O'Daniel (2005)	57 nurses (N=57)	Professions	7.5
Sliter, et al. [54]	2014	USA	n/a	Interpersonal conflict	Questionnaire, Interpersonal conflict at work scale (ICAWS) (Spector and Jex, 1998)	Nurses (N=172)	Profession<br>Culture	11
Veltman [55]	2007	USA	Labor and Delivery in 56 hospitals	Disruptive behaviors	Questionnaire, developed by Rosenstein and O'Daniel (2005)	Nurse managers (N=56)	Professions	7.5
McLemore [29]	2006	n/a	n/a	Workplace aggression	Interviews	Nurses (N=4)	Initiators	n/a
Riley and Manias [31]	2006	n/a – probably USA	OR, 3 hospitals	Tension and interpersonal conflicts	Ethnographic observations, group and individual interviews	OR nurses (N=11)	Situations	n/a
**All incivilities and all's point of view**								
Rehder, et al. [68]	2020	USA	Mixed, 16 hospitals in one healthcare system	Disruptive behaviors	Questionnaire, developed by the authors	Healthcare professionals (N=7923)	Profession<br><br>Culture	12
Chrouser and Partin [36]	2019	USA	OR in one academic medical training center	Disruptive behavior	Field notes from residency interviews	Medical students (N=42)	Profession<br>Initiators<br>Situations	n/a
Heslin, et al. [24]	2019	USA	Mixed, in one large tertiary medical academic center	Disruptive behavior	Reports on disruptive behaviors, from the perspective of the reporter and the involved party	Event-based analysis (N=314 event reports)	Professions<br>Situations	14
Keller, et al. [26]	2019	Switzerland	OR, two academic hospitals	Disruptive behaviors/tense communication	Observations (SO-DIC-OR) (Seelandt et al., 2014) and questionnaires developed by the authors	Event-based analysis (N=340 observed events)	Professions<br><br>Situations	13
Villafranca, et al. [39]	2019	Canada, US,	OR in different hospitals	Disruptive behavior	Questionnaire, developed by	Anesthesiologists,	Targets	11

		UK, Australia, NZ, India, Brazil, Other			Villafranca, Hamlin, Rodebaugh, Robinson, Jacobsohn (2017)	Nurses, Surgeons, other (technicians, med students) (N=7465)	Culture Professions Culture	
Bae, et al. [44]	2016	USA	Probably mixed, one urban academic medical center	Disruptive behavior	Questionnaire, John Hopkins disruptive behavior survey (JH-DCBS)	Nurses, midwifes, CRNAs, physician assistants, MDs (N=1559)	Targets Professions Situations Culture	10
Hamblin, et al. [22]	2016	USA	Probably mixed, in a large hospital system with 7 hospitals	Workplace violence	Retrospective chart analysis based on quantitative material	Perpetrators (N=185) for 199 violence incidents	Initiators Targets Professions	11
Berman-Kishony and Shvarts [33]	2015	Israel	Probably mixed, one medical center	Disruptive behavior	Questionnaire, developed by the authors based on focus groups and meetings	Nurses (n=76) and physicians (n=58)	Initiators Situations	9
Hamblin, et al. [32]	2015	USA	Probably mixed, in a large metropolitan hospital system with 7 hospitals	Workplace violence	Retrospective chart analysis based on qualitative material	Violence and incivility incidents for which a catalyst could be identified (N=135)	Professions Situations	n/a
Walrath, et al. [50]	2013	USA	Mixed, in one hospital	Disruptive behavior	Questionnaire	RN, MDs, affiliates (N=1559)	Professions	9
Rosenstein and Naylor [34]	2012	USA	ED, 20 different EDs	Disruptive behavior	Questionnaire, developed by the authors	Physician, nurses, secretaries or clerks, ED technicians (N=237)	Personality Professions Culture Situations	8
Rosenstein and O'Daniel [9]	2008	USA	Mixed, in 102 hospitals	Disruptive behavior	Questionnaire, developed by the authors	Physicians, nurses, administrative employees and others (N=4530)	Professions	7
Rosenstein and O'Daniel [51]	2005	USA	Mixed, in 50 hospitals	Disruptive behavior	Questionnaire, developed by the authors	RN, physicians, administrators (N=1509)	Professions	8

Lingard, et al. [15]	2002	n/a	OR in one teaching hospital	Tension	Ethnographic observation	All OR team members (N=n/a)	Situations	n/a

Note. [1]MERSQI scores are only available for quantitative studies

Table 2. Situational triggers of incivilities in healthcare teams

Authors	*Situations*	
Brewer, et al. [42]	More physician abuse associated with fewer nurses working than scheduled	*Workload*
Boateng and Adams [28]	If heavy work responsibilities, minority nurses reported conflicts about who did what (expertise)	*Work responsibilities*
Hamblin, et al. [32]	Work behavior: unprofessional behavior, duties and responsibilities, methods of care, poor performance.<br>Work organization: conflicts about tasks and procedures, organizational constraints, interdependence between the workers	*Communication/Teamwork,<br>Patient safety,<br>Work responsibilities<br>Organizational constraints*
Nemeth, et al. [80]	Most highly causal explanation was stress related to inadequate staffing or resources, followed by societal decline in civil behavior	*Workload*
Keller, et al. [13]	Organizational constraints predicted more incivility; no effect of quantitative workload	*Workload (no effect),<br>Organizational constraints*
Pattani, et al. [30]	Infrequent interactions	*Lack of familiarity*
Viotti, et al. [59]	Workload as a predictor of incivility only in the US but not in the Italian sample	*Workload (in one of the study samples)*
Berman-Kishony and Shvarts [33]	High workload is the second most frequent cause reported, followed by poor communication, distrust and disrespect	*Workload,<br>Communication/teamwork*
Budin, et al. [43]	Higher levels of verbal abuse perceived by nurses as associated with: Fewer nurses working than scheduled (staffing shortfalls), less perceived distributive and procedural justice, less promotional opportunities, more organizational constraints, higher quantitative workload	*Workload*
Cochran and Elder [27]	In the operating room, incivility was associated with: unfamiliar teams or trainees, something goes wrong during the operation, when there are differences in opinions with the surgeon while planning the operation	*Familiarity<br>Workload or patient safety*
Rosenstein and Naylor [34]	Delays, inadequate staffing and poor communication were rated less frequently than personality and attitudes	*Workload<br>Communication/teamwork*

For peer review only - http://bmjopen.bmj.com/site/about/guidelines.xhtml

Riley and Manias [31]	Time: questioning judgement time, controlling speed, estimating surgeon's time, different perceptions of time	*Time*
Elhoseny and Adel [60]	Workload as first root cause (reported by 35%), 15% reported compensation-related factors, Other: non work-related situations (12%)	*Workload*<br>*Non-wok related factors*
Bradley, et al. [49]	Doctors describing the situations in which they are rude: high workload, patient safety compromised, hierarchy	*Workload,*<br>*Patient safety*
Lingard, et al. [15]	Time, resources, roles, safety and sterility, situation control	*Communication/Teamwork,*<br>*Patient safety,*<br>*Time*
Bae, et al. [44]	Triggers of disruptive behaviors at the inter-individual level (e.g. questioning providers about care, lack of teamwork, staff diversity) and intrapersonal level (e.g. lack of competency, fatigue) related to experienced disruptive behaviors. Among nurses only (not physicians) organizational triggers (pressure from high volume, overload, unresolved issues unit culture) were also predictors of disruptive behaviors	*Workload,*<br>*Communication/teamwork*<br>*Patient safety*<br>*Fatigue*
Shetty, et al. [21]	Consultations with requests for investigations	*Request*
Heslin, et al. [24]	Patient factors mentioned as triggers (e.g.challenging anatomy), technical and environmental factors, organizational factors, stressors (individual or team)	*Workload*<br>*Communication/teamwork*
Chrouser and Partin [36]	Patient factors mentioned as triggers (e.g.challenging anatomy), technical and environmental factors, organizational factors, stressors (individual or team)	*Communication/teamwork*<br>*Organizational constraints*<br>*Task difficulty/stress*
Keller, et al. [26]	Collaboration and task related issues were clearly more frequent sources of tensions than relationship issues or disagreement about the task	*Communication/teamwork*<br>*Task difficulty/stress*
Rehder, et al. [68]	Disruptive behaviors correlated with poorer experienced teamwork, lower job satisfaction and lower perception of management	*Communication/teamwork*

Funding statement: This work was sponsored by a grant of the Swiss National Science Foundation, grant number P2NEP1 178574.

Conflict of interests: The authors report no conflict of interest

Data availability statement: All data relevant to the study are included in the article or uploaded as supplementary information

Authors' contributions:
Study design: Sandra Keller, Sarah Henrickson Parker and Steven Yule
Data analysis: Sandra Keller, Vivian Zagarese and Sarah Henrickson Parker
Drafting the work or critically revising it: Sandra Keller, Steven Yule, Vivian Zagarese, Sarah Henrickson Parker

Acknowledgements: We thank Rita McCandless and Erin M. Smith, Ph.D, for their technical support and advice about the library resources and giving us access to the full texts that could not be retrieved by the authors. We thank Franziska Tschan, PhD for her critical review of an earlier version of the manuscript.

Patient and public involvement statement: It was not appropriate or possible to involve patients or the public in the design, or conduct, or reporting, or dissemination plans of our research

Literature

1. Leape LL, Shore MF, Dienstag JL, et al. Perspective: a culture of respect, part 1: the nature and causes of disrespectful behavior by physicians. *Academic medicine* 2012;87(7):845-52.
2. Flin R. Rudeness at work: British Medical Journal Publishing Group, 2010.
3. Andersson LM, Pearson CM. Tit for tat? The spiraling effect of incivility in the workplace. *Academy of management review* 1999;24(3):452-71.
4. Shapiro J. Confronting unprofessional behaviour in medicine: British Medical Journal Publishing Group, 2018.
5. Rosenstein AH, O'Daniel M. Impact and implications of disruptive behavior in the perioperative arena. *Journal of the American College of Surgeons* 2006;203(1):96-105.
6. Salazar MJB, Minkoff H, Bayya J, et al. Influence of surgeon behavior on trainee willingness to speak up: a randomized controlled trial. *Journal of the American College of Surgeons* 2014;219(5):1001-07.
7. Riskin A, Erez A, Foulk TA, et al. The impact of rudeness on medical team performance: a randomized trial. *Pediatrics* 2015;136(3):487-95.
8. Porath CL, Pearson CM. Emotional and behavioral responses to workplace incivility and the impact of hierarchical status. *Journal of Applied Social Psychology* 2012;42(S1):E326-E57.
9. Rosenstein AH, O'Daniel M. A survey of the impact of disruptive behaviors and communication defects on patient safety. *The Joint Commission Journal on Quality and Patient Safety* 2008;34(8):464-71.
10. Small CR, Porterfield S, Gordon G. Disruptive behavior within the workplace. *Applied Nursing Research* 2015;28(2):67-71. doi: 10.1016/j.apnr.2014.12.002
11. Hershcovis MS, Barling J. Towards a multi-foci approach to workplace aggression: A meta-analytic review of outcomes from different perpetrators. *Journal of Organizational Behavior* 2010;31(1):24-44.
12. Kaiser JA. The relationship between leadership style and nurse-to-nurse incivility: turning the lens inward. *J Nurs Manag* 2017;25(2):110-18. doi: 10.1111/jonm.12447
13. Keller R, Krainovich-Miller B, Budin W, et al. Predictors of nurses' experience of verbal abuse by nurse colleagues. *Nurs Outlook* 2018;66(2):190-203. doi: 10.1016/j.outlook.2017.10.006 [published Online First: 2018/04/25]
14. Mullan CP, Shapiro J, McMahon GT. Interns' experiences of disruptive behavior in an academic medical center. *Journal of graduate medical education* 2013;5(1):25-30. doi: 10.4300/jgme-d-12-00025.1 [published Online First: 2014/01/10]
15. Lingard L, Reznick R, Espin S, et al. Team communications in the operating room: talk patterns, sites of tension, and implications for novices. *Academic Medicine* 2002;77(3):232-37.
16. Oja KJ. Incivility and Professional Comportment in Critical Care Nurses. *AACN advanced critical care* 2017;28(4):345-50.
17. Abdollahzadeh F, Asghari E, Doshmangir L, et al. Workplace Incivility as an Extensively Used, But Seldom Defined Concept in Nursing. *Nurs Midwifery Stud* 2017;6(2):e41029.
18. Bambi S, Foà C, De Felippis C, et al. Workplace incivility, lateral violence and bullying among nurses. A review about their prevalence and related factors. *Acta bio-medica: Atenei Parmensis* 2018;89(Suppl 6):51.
19. Moher D, Shamseer L, Clarke M, et al. Preferred reporting items for systematic review and meta-analysis protocols (PRISMA-P) 2015 statement. *Systematic reviews* 2015;4(1):1.
20. Reed DA, Cook DA, Beckman TJ, et al. Association between funding and quality of published medical education research. *Jama* 2007;298(9):1002-09.
21. Shetty AL, Vaghasiya M, Boddy R, et al. Perceived incivility during emergency department phone consultations. *Emerg Med Australas* 2016;28(3):256-61. doi: 10.1111/1742-6723.12564

For peer review only - http://bmjopen.bmj.com/site/about/guidelines.xhtml

22. Hamblin LE, Essenmacher L, Ager J, et al. Worker-to-Worker Violence in Hospitals Perpetrator Characteristics and Common Dyads. *Workplace Health Saf* 2016;64(2):51-56. doi: 10.1177/2165079915608856
23. Goettler CE, Butler TS, Shackleford P, et al. Physician Behavior: Not Ready for ‘Never'land. *The American Surgeon* 2011;77(12):1600-05.
24. Heslin MJ, Singletary BA, Benos KC, et al. Is Disruptive Behavior Inherent to the Surgeon or the Environment? Analysis of 314 Events at a Single Academic Medical Center. *Annals of surgery* 2019;270(3):463-72.
25. Finlayson AJR, Dietrich MS, Neufeld R, et al. Restoring professionalism: the physician fitness-for-duty evaluation. *Gen Hosp Psych* 2013;35(6):659-63. doi: 10.1016/j.genhosppsych.2013.06.009
26. Keller S, Tschan F, Semmer NK, et al. “Disruptive behavior” in the operating room: A prospective observational study of triggers and effects of tense communication episodes in surgical teams. *PLoS One* 2019;14(12)
27. Cochran A, Elder WB. A model of disruptive surgeon behavior in the perioperative environment. *Journal of the American College of Surgeons* 2014;219(3):390-98. doi: 10.1016/j.jamcollsurg.2014.05.011
28. Boateng GO, Adams TL. "Drop dead ... I need your job": An exploratory study of intra-professional conflict amongst nurses in two Ontario cities. *Soc Sci Med* 2016;155:35-42. doi: 10.1016/j.socscimed.2016.02.045
29. McLemore MR. CJON reporter. Workplace aggression: beginning a dialogue. *Clinical Journal of Oncology Nursing* 2006;10(4):455-56. doi: 10.1188/06.CJON.455-456
30. Pattani R, Ginsburg S, Johnson AM, et al. Organizational factors contributing to incivility at an academic medical center and systems-based solutions: a qualitative study. *Academic Medicine* 2018;93(10):1569.
31. Riley R, Manias E. Governing time in operating rooms. *Journal of clinical nursing* 2006;15(5):546-53.
32. Hamblin LE, Essenmacher L, Upfal MJ, et al. Catalysts of worker-to-worker violence and incivility in hospitals. *Journal of Clinical Nursing* 2015;24(17/18):2458-67. doi: 10.1111/jocn.12825
33. Berman-Kishony T, Shvarts S. Universal versus tailored solutions for alleviating disruptive behavior in hospitals. *Isr J Health Policy Res* 2015;4:12. doi: 10.1186/s13584-015-0018-7
34. Rosenstein AH, Naylor B. Incidence and impact of physician and nurse disruptive behaviors in the emergency department. *The Journal of emergency medicine* 2012;43(1):139-48.
35. Bansal AS. Disruptive behaviour amongst doctors, myth or reality? *Journal of Evolution of Medical and Dental Sciences* 2014;3(2)
36. Chrouser KL, Partin MR. Intraoperative Disruptive Behavior: The Medical Student's Perspective. *Journal of surgical education* 2019
37. Sellers KF, Millenbach L, Ward K, et al. The Degree of Horizontal Violence in RNs Practicing in New York State. *J Nurs Adm* 2012;42(10):483-87. doi: 10.1097/NNA.0b013e31826a208f
38. Alshehry AS, Alquwez N, Almazan J, et al. Workplace incivility and its influence on professional quality of life among nurses from multicultural background: A cross-sectional study. *Journal of clinical nursing* 2019;28(13-14):2553-64.
39. Villafranca A, Hiebert B, Hamlin C, et al. Prevalence and predictors of exposure to disruptive behaviour in the operating room. *Canadian Journal of Anesthesia/Journal canadien d'anesthésie* 2019;66(7):781-94.
40. Ruvalcaba JG, Welch S, Carlisle J. ESL Versus Non-ESL Nursing Students' Perceptions of Incivility in the Clinical Setting. *Journal of Nursing Education* 2018;57(12):720-26.
41. Heydari A, Rad M, Rad M. Evaluating the Incivility between Staff Nurses and Matrons Employed in Iran. *Acta Fac Medicae Naiss* 2015;32(2):137-46. doi: 10.1515/afmnai-2015-0014
42. Brewer CS, Kovner CT, Obeidat RF, et al. Positive work environments of early-career registered nurses and the correlation with physician verbal abuse. *Nurs Outlook* 2013;61(6):408-16. doi: 10.1016/j.outlook.2013.01.004

For peer review only - http://bmjopen.bmj.com/site/about/guidelines.xhtml

43. Budin WC, Brewer CS, Chao YY, et al. Verbal Abuse From Nurse Colleagues and Work Environment of Early Career Registered Nurses. *J Nurs Scholarsh* 2013;45(3):308-16. doi: 10.1111/jnu.12033
44. Bae SH, Dang D, Karlowicz KA, et al. Triggers Contributing to Health Care Clinicians' Disruptive Behaviors. *Journal of patient safety* 2016 doi: 10.1097/pts.0000000000000288 [published Online First: 2016/11/05]
45. Chang HE, Park MY, Jang H, et al. Relationships among demands at work, aggression, and verbal abuse among registered nurses in South Korea. *Nurs Outlook* 2019;67(5):567-77.
46. Birks M, Cant RP, Budden LM, et al. Uncovering degrees of workplace bullying: A comparison of baccalaureate nursing students' experiences during clinical placement in Australia and the UK. *Nurse Educ Pract* 2017;25:14-21. doi: 10.1016/j.nepr.2017.04.011
47. Budden LM, Birks M, Cant R, et al. Australian nursing students' experience of bullying and/or harassment during clinical placement. *Collegian* 2017;24(2):125-33. doi: 10.1016/j.colegn.2015.11.004
48. Minton C, Birks M, Cant R, et al. New Zealand nursing students' experience of bullying/harassment while on clinical placement: A cross-sectional survey. *Collegian* 2018;25(6):583-89.
49. Bradley V, Liddle S, Shaw R, et al. Sticks and stones: investigating rude, dismissive and aggressive communication between doctors. *Clin Med* 2015;15(6):541-45. doi: 10.7861/clinmedicine.15-6-541
50. Walrath JM, Dang D, Nyberg D. An Organizational Assessment of Disruptive Clinician Behavior Findings and Implications. *J Nurs Care Qual* 2013;28(2):110-21. doi: 10.1097/NCQ.0b013e318270d2ba
51. Rosenstein AH, O'Daniel M. Original Research: Disruptive Behavior and Clinical Outcomes: Perceptions of Nurses and Physicians: Nurses, physicians, and administrators say that clinicians' disruptive behavior has negative effects on clinical outcomes. *AJN The American Journal of Nursing* 2005;105(1):54-64.
52. Addison K, Luparell S. Rural Nurses' Perception of Disruptive Behaviors and Clinical Outcomes: A Pilot Study. *Online Journal of Rural Nursing & Health Care* 2014;14(1):66-82. doi: 10.14574/ojrnhc.v14i1.300
53. Elmblad R, Kodjebacheva G, Lebeck L. Workplace Incivility Affecting CRNAs: A Study of Prevalence, Severity, and Consequences With Proposed Interventions. *AANA Journal* 2014;82(6):437-45.
54. Sliter M, Boyd E, Sinclair R, et al. Inching Toward Inclusiveness: Diversity Climate, Interpersonal Conflict and Well-Being in Women Nurses. *Sex Roles* 2014;71(1-2):43-54. doi: 10.1007/s11199-013-0337-5
55. Veltman LL. Disruptive behavior in obstetrics: a hidden threat to patient safety. *American Journal of Obstetrics and Gynecology* 2007;196(6):587. e1-87. e5.
56. Lewis PS, Malecha A. The impact of workplace incivility on the work environment, manager skill, and productivity. *J Nurs Adm* 2011;41(1):41-47.
57. Klingberg K, Gadelhak K, Jegerlehner SN, et al. Bad manners in the Emergency Department: Incivility among doctors. *PLoS One* 2018;13(3):11. doi: 10.1371/journal.pone.0194933
58. Layne DM, Anderson E, Henderson S. Examining the presence and sources of incivility within nursing. *J Nurs Manag* 2019;27(7):1505-11.
59. Viotti S, Converso D, Hamblin LE, et al. Organisational efficiency and co-worker incivility: A cross-national study of nurses in the USA and Italy. *J Nurs Manag* 2018 doi: 10.1111/jonm.12587 [published Online First: 2018/01/11]
60. Elhoseny TA, Adel A. Disruptive physician behaviors and their impact on patient care in a health insurance hospital in Alexandria, Egypt. *Journal of the Egyptian Public Health Association* 2016;91(2):80-85.
61. Smith JG, Morin KH, Lake ET. Association of the nurse work environment with nurse incivility in hospitals. *J Nurs Manag* 2018;26(2):219-26. doi: 10.1111/jonm.12537

For peer review only - http://bmjopen.bmj.com/site/about/guidelines.xhtml

62. Minton C, Birks M. “You can't escape it”: Bullying experiences of New Zealand nursing students on clinical placement. *Nurse education today* 2019;77:12-17.
63. Alkaabi O, Wong C. Relationships among authentic leadership, manager incivility and trust in the manager. *Leadership in Health Services* 2019
64. Kramer M, Schmalenberg C. Magnet hospitals: Part I. Institutions of excellence. *The Journal of nursing administration* 1988;18(1):13-24.
65. Association AN. ANCC Magnet Recognition Program® [Available from: https://www.nursingworld.org/organizational-programs/magnet/ accessed November 1st 2019.
66. Arslan Yürümezoğlu H, Kocaman G. Structural empowerment, workplace incivility, nurses' intentions to leave their organisation and profession: A path analysis. *J Nurs Manag* 2019;27(4):732-39.
67. Tikva SS, Kluger AN, Lerman Y. Disruptive behaviors among nurses in Israel–association with listening, wellbeing and feeling as a victim: a cross-sectional study. *Isr J Health Policy Res* 2019;8(1):1-9.
68. Rehder KJ, Adair KC, Hadley A, et al. Associations Between a New Disruptive Behaviors Scale and Teamwork, Patient Safety, Work-Life Balance, Burnout, and Depression. *The Joint Commission Journal on Quality and Patient Safety* 2020;46(1):18-26.
69. Villafranca A, Hamlin C, Enns S, et al. Disruptive behaviour in the perioperative setting: a contemporary review. *Canadian Journal of Anesthesia/Journal canadien d'anesthésie* 2017;64(2):128-40.
70. Müller P, Tschan F, Keller S, et al. Assessing Perceptions of Teamwork Quality Among Perioperative Team Members. *AORN journal* 2018;108(3):251-62.
71. Harris WC, Usseglio J, Chapman-Rodriguez R, et al. A Scoping Review of Validated Tools to Measure Incivility in Healthcare Settings. *JONA: The Journal of Nursing Administration* 2019;49(9):447-53.
72. Jehn KA. A qualitative analysis of conflict types and dimensions in organizational groups. *Administrative science quarterly* 1997:530-57.
73. Bochatay N, Bajwa NM, Cullati S, et al. A multilevel analysis of professional conflicts in health care teams: insight for future training. *Academic Medicine* 2017;92(11S):S84-S92.
74. Katz JD. Conflict and its resolution in the operating room. *Journal of clinical anesthesia* 2007;19(2):152-58.
75. Hills DJ. Defining and classifying aggression and violence in health care work. *Collegian* 2018;25(6):607-12.
76. Wing T, Regan S, Spence Laschinger HK. The influence of empowerment and incivility on the mental health of new graduate nurses. *J Nurs Manag* 2015;23(5):632-43.
77. Babenko-Mould Y, Laschinger HK. Effects of incivility in clinical practice settings on nursing student burnout. *International journal of nursing education scholarship* 2014;11(1):145-54.
78. Bansal AS. DISRUPTIVE BEHAVIOUR AMONGST DOCTORS, MYTH OR REALITY? *Journal of Evolution of Medical and Dental Sciences-Jemds* 2014;3(2):399-406. doi: 10.14260/jemds/2014/1841
79. Jones D, Madden C, Miles C. Privileged access by irrelevant speech to short-term memory: The role of changing state. *The Quarterly Journal of Experimental Psychology* 1992;44(4):645-69.
80. Nemeth LS, Stanley KM, Martin MM, et al. Lateral Violence in Nursing Survey: Instrument Development and Validation. *Healthcare* 2017;5(3):12. doi: 10.3390/healthcare5030033

For peer review only - http://bmjopen.bmj.com/site/about/guidelines.xhtml

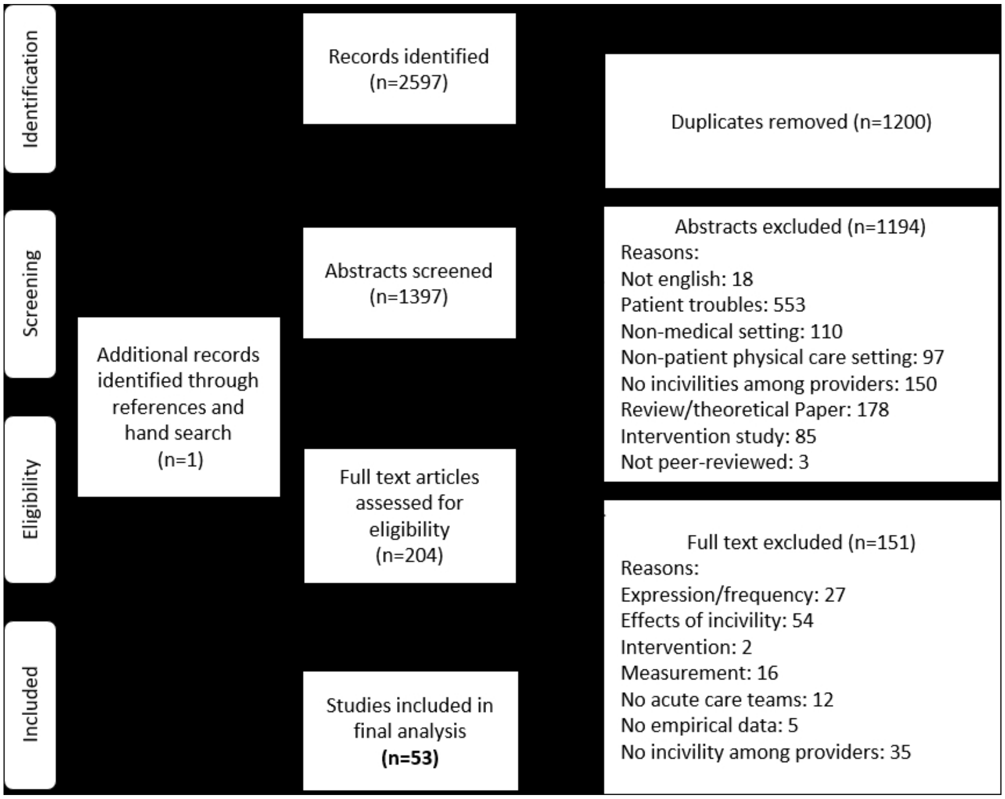
Identification
Screening
Eligibility
Included
Records identified
(n=2597)
Duplicates removed (n=1200)
Abstracts screened
(n=1397)
Abstracts excluded (n=1194)
Reasons:
Not english: 18
Patient troubles: 553
Non-medical setting: 110
Non-patient physical care setting: 97
No incivilities among providers: 150
Review/theoretical Paper: 178
Intervention study: 85
Not peer-reviewed: 3
Additional records identified through references and hand search
(n=1)
Full text articles assessed for eligibility
(n=204)
Full text excluded (n=151)
Reasons:
Expression/frequency: 27
Effects of incivility: 54
Intervention: 2
Measurement: 16
No acute care teams: 12
No empirical data: 5
No incivility among providers: 35
Studies included in final analysis
(n=53)

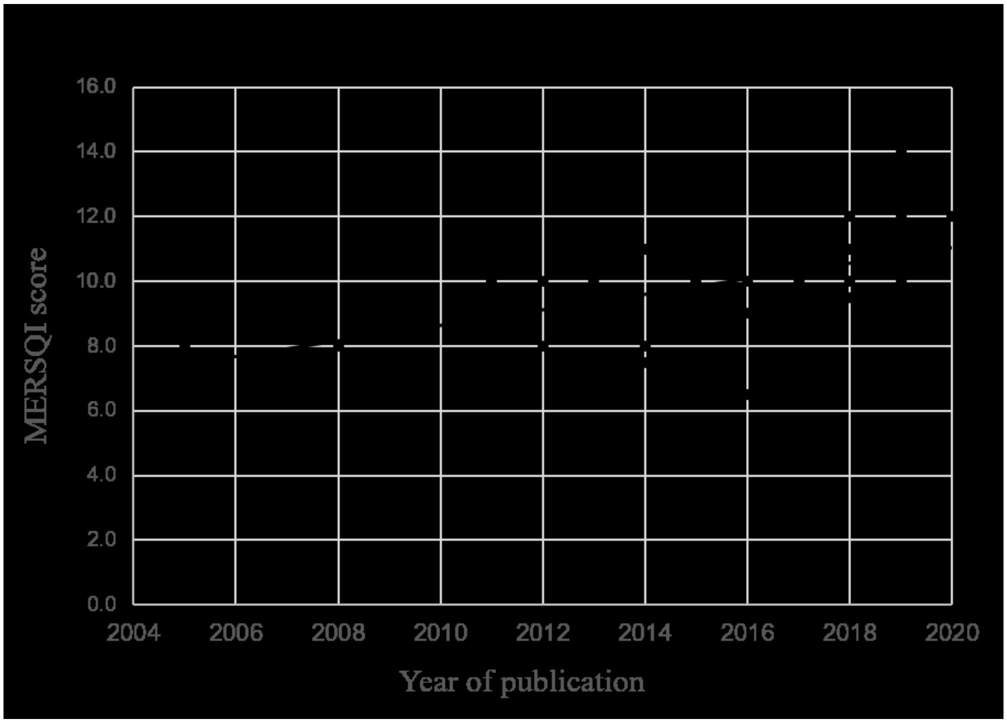
MERSQI score
16.0
14.0
12.0
10.0
8.0
6.0
4.0
2.0
0.0
2004
2006
2008
2010
2012
2014
2016
2018
2020
Year of publication

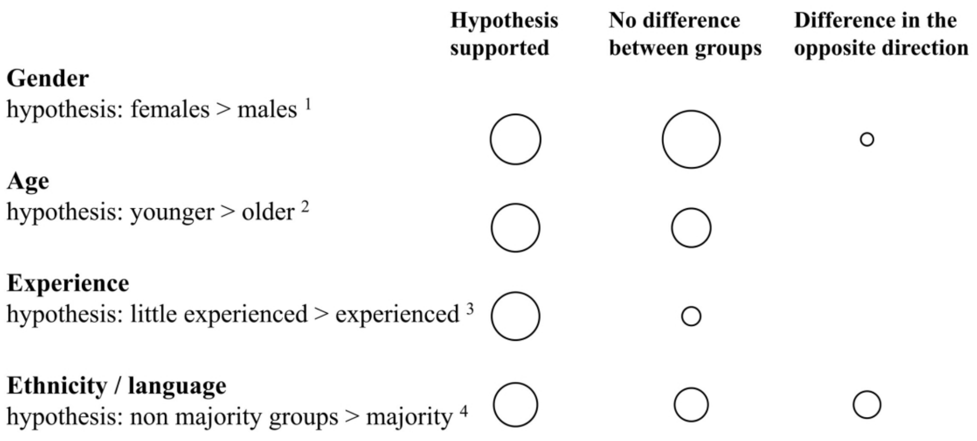

Note. The size of the bubble represents the number of studies included that support the hypothesis, showed no differences between group respectively showed differences in the opposite direction

[1] Female healthcare professionals may experience more incivility

[2] Younger healthcare professionals may experience more incivility

[3] Healthcare professionals who have less work experienced may experience more incivility

[4] Healhcare professionals who belong to a visible ethnic minority group or are no native speaker may experience more incivility

199x152mm (300 x 300 DPI)

Additional Material, Table 1: Search strategy used on Medline

**Step 1: Search in a mesh term and title and abstract**		
**Concept of interest**		**Settings of interest**
MeSH Term	***Combined with***	***At least one of the following terms in the Title or Abstract***
incivility	(“and”)	hospital
		operating room
		operating theatre
		Surgery
		intensive care unit
		ICU
		medical team
		physician
		doctor
		nurse
		anesthetist
		anesthesiologist
		anesthesia
		emergency department
		peri-operative
		obstetrics
		gynecology
		CRNA
		pediatrician
		surgeon
		resident
		medical student
		internal medicine
		palliative
		otorhinolaryngology
**Step 2: Search in title and abstract**		
**Concept of interest**		**Settings of interest**
At least one of the following terms in the Title or Abstract	***Combined with (“and”)***	***At least one of the following terms in the Title or Abstract***
incivility		hospital
rudeness		operating room
disruptive behavior		operating theatre
unprofessional behavior		Surgery
interpersonal tension		intensive care unit
		ICU
		medical team

For peer review only

Additional Material, Table 1: Search strategy used on Medline

physician
doctor
nurse
anesthetist
anesthesiologist
anesthesia
emergency department
peri-operative
obstetrics
gynecology
CRNA
pediatrician
surgeon
resident
medical student
internal medicine
palliative
otorhinolaryngology

For peer review only

Additional material, Table 2: details of MERSQI scores

	**design**	**sampling**		**type of data**	**Validity of instrument**			**Data analysis**		**Types of outcome measured**				**Total score**
Author		*Institutions studied*	*Response rate score*		*Internal structure*	*Content*	*Relationship to other variables*	*Appropriateness*	*Complexity*	*Satisfaction, attitudes, perception*	*Knowledge, skills*	*Behaviors*	*Patient/health care outcome*	
Addison and Luparell (2014)	1	1	0.5	1	0	1	0	1	1	1	0	0	0	**7.5**
Alkaabi and Wong (2019)	1	1.5	0.5	1	1	1	1	1	2	1	0	0	0	**11**
Alshehry et al. (2019)	1	1	1.0	1	1	1	1	1	2	1	0	0	0	**11**
Arslan Yürümezoğlu and Kocaman (2019)	1	1	1.0	1	1	1	1	1	2	1	0	0	0	**11**
Bae et al. (2016)	1	1.5	0.5	1	1	1	0	1	2	1	0	0	0	**10**
Bansal (2014)	1	0.5	0.5	1	1	1	0	1	1	1	0	0	0	**8**
Berman-Kishony and Shvarts (2015)	1	0.5	0.5	1	0	1	1	1	2	1	0	0	0	**9**
Birks et al. (2017)	1	1.5	0.5	1	1	1	0	1	2	1	0	0	0	**10**
Bradley et al. (2015)	1	1.5	0.5	1	0	0	0	1	1	1	0	0	0	**7**
Brewer et al. (2013)	1	1.5	1.0	1	1	1	0	1	1	1	0	0	0	**9.5**
Budden et al. (2017)	1	1.5	0.5	1	1	1	0	1	2	1	0	0	0	**10**
Budin et al. (2013)	1	1.5	1.0	1	1	1	1	0	2	1	0	0	0	**10.5**
Chang et al. (2019)	1	1.5	1.5	1	1	1	1	1	2	1	0	0	0	**12**
Elhoseny and Adel (2016)	1	0.5	1.0	1	0	0	0	1	1	1	0	0	0	**6.5**
Elmblad et al. (2014)	1	1.5	0.5	1	1	1	1	1	2	1	0	0	0	**11**

For peer review only

Additional material, Table 2: details of MERSQI scores

Finlayson et al. (2013)	1	1.5	0.5	3	0	1	1	1	2	0	0	2	0	**13**
Goettler et al. (2011)	1	0.5	1.5	1	0	1	0	1	2	1	0	0	0	**9**
Hamblin et al. (2016)	1	1.5	1.5	1	0	1	0	1	2	1	0	0	0	**10**
Heslin et al. (2019)	1	0.5	1.5	3	1	1	1	1	2	1	0	1	0	**14**
Heydari et al. (2015)	1	1.5	1.5	1	1	1	0	0	2	1	0	0	0	**10**
Kaiser (2017)	1	0.5	0.5	1	1	1	1	1	2	1	0	0	0	**10**
Keller et al. (2018)	1	1.5	1.5	1	1	1	1	1	2	1	0	0	0	**12**
Keller et al. (2019)	1	1	1.5	3	1	0	1	1	2	1	0	1	0	**13**
Klingberg et al. (2018)	1	0.5	1.0	1	0	1	1	1	2	1	0	0	0	**9.5**
Layne et al. (2019)	1	0.5	0.5	1	1	1	0	1	2	1	0	0	0	**9**
Lewis and Malecha (2011)	1	1.5	0.5	1	1	1	0	1	2	1	0	0	0	**10**
Minton et al. (2018)	1	1.5	0.5	1	1	1	0	1	2	1	0	0	0	**10**
Mullan et al. (2013)	1	1.5	1.5	1	0	1	0	1	2	1	0	0	0	**10**
Nemeth et al. (2017)	1	0.5	0.5	1	1	1	0	1	2	1	0	0	0	**9**
Rehder et al. (2020)	1	1.5	1.5	1	1	1	1	1	2	1	0	0	0	**12**
Rosenstein and Naylor (2012)	1	1.5	0.5	1	0	1	0	1	1	1	0	0	0	**8**
Rosenstein and O'Daniel (2005)	1	1.5	0.5	1	0	1	0	1	1	1	0	0	0	**8**
Rosenstein and O'Daniel (2008)	1	1.5	0.5	1	0	1	0	1	0	1	0	0	0	**7**

For peer review only

Additional material, Table 2: details of MERSQI scores

Ruvalcaba et al. (2018)	1	1.5	0.5	1	1	1	0	1	2	1	0	0	0	**10**
Sellers et al. (2012)	1	1.5	0.5	1	1	1	0	1	2	1	0	0	0	**10**
Shetty et al. (2016)	1	1.5	0.5	1	0	1	0	1	2	1	0	0	0	**9**
Sliter et al. (2014)	1	1.5	0.5	1	1	1	1	1	2	1	0	0	0	**11**
Small et al. (2015)	1	1.5	0.5	1	0	1	0	1	2	1	0	0	0	**9**
Smith et al. (2018)	1	1.5	0.5	1	1	1	1	1	2	1	0	0	0	**11**
Tikva et al. (2019)	1	1.5	0.5	1	1	0	1	1	2	1	0	0	0	**10**
Veltman (2007)	1	1.5	1.0	1	0	0	0	1	1	1	0	0	0	**7.5**
Villafranca et al. (2019)	1	1.5	0.5	1	1	1	1	1	2	1	0	0	0	**11**
Viotti et al. (2018)	1	1.5	0.5	1	1	1	1	1	2	1	0	0	0	**11**
Walrath et al. (2013)	1	0.5	0.5	1	1	1	0	1	2	1	0	0	0	**9**

For peer review only

PRISMA 2009 Checklist

Section/topic	#	Checklist item	Reported on page #
**TITLE**			
Title	1	Identify the report as a systematic review, meta-analysis, or both.	1
**ABSTRACT**			
Structured summary	2	Provide a structured summary including, as applicable: background; objectives; data sources; study eligibility criteria, participants, and interventions; study appraisal and synthesis methods; results; limitations; conclusions and implications of key findings; systematic review registration number.	2
**INTRODUCTION**			
Rationale	3	Describe the rationale for the review in the context of what is already known.	4
Objectives	4	Provide an explicit statement of questions being addressed with reference to participants, interventions, comparisons, outcomes, and study design (PICOS).	4
**METHODS**			
Protocol and registration	5	Indicate if a review protocol exists, if and where it can be accessed (e.g., Web address), and, if available, provide registration information including registration number.	
Eligibility criteria	6	Specify study characteristics (e.g., PICOS, length of follow-up) and report characteristics (e.g., years considered, language, publication status) used as criteria for eligibility, giving rationale.	5
Information sources	7	Describe all information sources (e.g., databases with dates of coverage, contact with study authors to identify additional studies) in the search and date last searched.	5
Search	8	Present full electronic search strategy for at least one database, including any limits used, such that it could be repeated.	See additional Material Table 1
Study selection	9	State the process for selecting studies (i.e., screening, eligibility, included in systematic review, and, if applicable, included in the meta-analysis).	5-6
Data collection process	10	Describe method of data extraction from reports (e.g., piloted forms, independently, in duplicate) and any processes for obtaining and confirming data from investigators.	5
Data items	11	List and define all variables for which data were sought (e.g., PICOS, funding sources) and any assumptions and simplifications made.	
Risk of bias in individual studies	12	Describe methods used for assessing risk of bias of individual studies (including specification of whether this was done at the study or outcome level), and how this information is to be used in any data synthesis.	5
Summary measures	13	State the principal summary measures (e.g., risk ratio, difference in means).	6

For peer review only

PRISMA 2009 Checklist

Synthesis of results	14	Describe the methods of handling data and combining results of studies, if done, including measures of consistency (e.g., I^2) for each meta-analysis.	6

Section/topic	#	Checklist item	Reported on page #
Risk of bias across studies	15	Specify any assessment of risk of bias that may affect the cumulative evidence (e.g., publication bias, selective reporting within studies).	
Additional analyses	16	Describe methods of additional analyses (e.g., sensitivity or subgroup analyses, meta-regression), if done, indicating which were pre-specified.	
**RESULTS**			
Study selection	17	Give numbers of studies screened, assessed for eligibility, and included in the review, with reasons for exclusions at each stage, ideally with a flow diagram.	6
Study characteristics	18	For each study, present characteristics for which data were extracted (e.g., study size, PICOS, follow-up period) and provide the citations.	17
Risk of bias within studies	19	Present data on risk of bias of each study and, if available, any outcome level assessment (see item 12).	Additional Material Table 2
Results of individual studies	20	For all outcomes considered (benefits or harms), present, for each study: (a) simple summary data for each intervention group (b) effect estimates and confidence intervals, ideally with a forest plot.	
Synthesis of results	21	Present results of each meta-analysis done, including confidence intervals and measures of consistency.	
Risk of bias across studies	22	Present results of any assessment of risk of bias across studies (see Item 15).	Additional Material Table 2
Additional analysis	23	Give results of additional analyses, if done (e.g., sensitivity or subgroup analyses, meta-regression [see Item 16]).	
**DISCUSSION**			
Summary of evidence	24	Summarize the main findings including the strength of evidence for each main outcome; consider their relevance to key groups (e.g., healthcare providers, users, and policy makers).	8-13
Limitations	25	Discuss limitations at study and outcome level (e.g., risk of bias), and at review-level (e.g., incomplete retrieval of identified research, reporting bias).	15
Conclusions	26	Provide a general interpretation of the results in the context of other evidence, and implications for future research.	16
**FUNDING**			

For peer review only

For peer review only - http://bmjopen.bmj.com/site/about/guidelines.xhtml

PRISMA 2009 Checklist

Funding	27	Describe sources of funding for the systematic review and other support (e.g., supply of data); role of funders for the systematic review.	

From: Moher D, Liberati A, Tetzlaff J, Altman DG, The PRISMA Group (2009). Preferred Reporting Items for Systematic Reviews and Meta-Analyses: The PRISMA Statement. PLoS Med 6(6): e1000097. doi:10.1371/journal.pmed1000097

For more information, visit: **www.prisma-statement.org**.

For peer review only

For peer review only - http://bmjopen.bmj.com/site/about/guidelines.xhtml
